# An ER-IMC bridge protein TgVPS13A and an IMC-resident scramblase TgDAT1 drive daughter budding in *Toxoplasma gondii*

**DOI:** 10.1371/journal.ppat.1013865

**Published:** 2026-06-18

**Authors:** Lin Zhao, Jiawen Fu, Swaroop Peddiraju, Heming Chen, Keqin Huang, Ying Zhang, Nishith Gupta, Qian Jiang, Honglin Jia

**Affiliations:** 1 State Key Laboratory of Animal Disease Control and Prevention, Harbin Veterinary Research Institute, Chinese Academy of Agricultural Sciences, Harbin, China; 2 Intracellular Parasite Education and Research Labs (iPEARL), Department of Biological Sciences, Birla Institute of Technology and Science, Pilani (BITS Pilani), Hyderabad, India; UTSW: The University of Texas Southwestern Medical Center, UNITED STATES OF AMERICA

## Abstract

All alveolates including apicomplexan parasites contain an inner membrane complex (IMC) underneath the plasma membrane. The IMC is synthesized *de novo* during asexual replication (including endodyogeny, endopolygeny, and schizogony) and serves as a crucial scaffold for supporting cytoskeletal structures and the glideosome machinery for parasite locomotion. However, the mechanism(s) underlying the membrane biogenesis in the IMC are not well understood. Using a clinically-relevant and globally-prevalent pathogenic protist model, *Toxoplasma gondii*, we identified the TgVPS13A bridging the IMC to the endoplasmic reticulum (ER) – the major site of phospholipid synthesis. The multi-modular TgVPS13A plays a crucial role in the IMC biogenesis, functioning in concert with an ER-resident VAP protein (TgVAP) and a novel lipid scramblase (TgDAT1). TgDAT1 is recruited for the progeny formation sites during the early stages of budding. Conditional depletion of TgVPS13A, TgDAT1 or TgVAP results in collapse of the inner membrane complex, leading to parasite death, as visualized by endodyogeny-specific organelle markers. GFP-Lact-C2, a biosensor of phosphatidylserine and phosphatidylthreonine lipids made in the ER and enriched in the IMC, also mislocalizes upon protein depletion. In conclusion, we propose that TgVPS13A, together with TgVAP and TgDAT1, bridge the ER and IMC and mediate the inter-organelle transport of lipids, thus contributing to the organelle biogenesis and daughter budding in *T. gondii.*

## Author summary

The inner membrane complex (IMC) in apicomplexan parasites is essential for maintaining the structural stability of the parasite, as well as for its budding and motility. The IMC is composed of flattened vesicles, alveolins, and microtubules. However, the mechanism behind the biogenesis of these flattened vesicles remains unclear. In this study, we provide evidence that a bridge-like protein TgVPS13A, together with TgVAP and TgDAT1 drive the biogenesis of the IMC alveoli. Phenotypic analysis indicates that the absence of any individual of these proteins disrupts the biogenesis of daughter IMCs, which in turn affects the budding of daughter parasites. We also demonstrated that TgDAT1 functions as a scramblase. Based on our findings, we propose a model in which the TgVPS13A, functioning in concert with TgVAP and TgDAT1 mediates lipid transport from the ER to the nascent IMC to drive the expansion of the IMC membrane and the budding of daughter parasites in *T. gondii*. Furthermore, TgDAT1 may facilitate the transfer of lipids from the outer leaflet to the inner leaflet of the IMC sacs, promoting a balanced composition of the IMC membrane components.

## Introduction

Apicomplexa are obligate intracellular parasites that pose significant health risks to humans and animals. Alongside ciliates and dinoflagellates, these protists belong to the superphylum Alveolata, which is hallmarked by a unique structure comprising membrane vesicles (alveoli, cisternae) situated just underneath the plasma membrane. In Apicomplexa, this membrane structure is referred to as the inner membrane complex (IMC). The IMC together with the plasma membrane forms the pellicle of the parasites, which harbors the actin-myosin motors and other locomotion-related protein machinery cumulatively termed as Glideosome. In *T. gondii*, the IMC is made up of flattened vesicles, networks of alveolin, and sub-membranous microtubules. This cytoskeletal structure is essential for the parasite’s replication, movement, and invasion of host cells [1]. The IMC begins from an apical polar ring (APR), and the apical region above the APR is encapsulated by the plasma membrane. The upper part of the IMC consists of a single conical IMC vesicle, while the structure at the base is known as the basal complex. Vesicles within the IMC are connected by longitudinal and transverse suture proteins [2]. During the progeny budding (endodyogeny), the IMC is made *de novo*. However, little is known about the mechanisms that govern the biosynthesis of the flattened vesicles within the IMC.

Membrane expansion needs a continuous supply of bulk lipids and transport from the source organelles, such as ER or lipid droplets (LDs). In other eukaryotes, lipid transfer proteins (LTPs) are proven to accomplish this process at the membrane contact sites (MCSs), where two organelles are proximal so that a single protein can span the entire distance between membranes to transfer lipids [3]. MCSs are considered to play a central role in non-vesicular lipid transport. Currently, ten members of LTPs have been identified, including VPS13 (A-D), ATG2 (A-B) and BLTPs (1, 2, 3A-B) [4]. VPS13 is a new class of LTPs that emerged recently. While several species, including plants and protists, contain multiple VPS13 genes, yeast possesses a single VPS13 protein. This family of proteins appears at multiple MCSs to mediate lipid transport in a way similar to ATG2 by forming a hydrophobic channel at MCSs [5–8]. Fragments of VPS13 were shown to bind multiple lipids and to transfer lipids between artificial liposomes [7]. For instance, human VPS13A resides at the MCSs between ER and mitochondria, endosome/lysosomes, lipid droplet (LDs) [7,9], or plasma membrane [10], VPS13B is localized at the interface of Golgi cisternae [11] or between ER exit site (ERES) and the Golgi [12], and VPS13C is localized at the MCS of the ER and endo/lysosomes [13], whereas VPS13D localizes at between the ER and mitochondria or peroxisomes [14].

The N-terminal region of VPS13 proteins is responsible for binding to adaptors at the donor organelle, which supply lipids. In contrast, the adaptors located at the opposing membrane interact with either the VAB domain or the C-terminal region of VPS13. For example, human VPS13 interacts with VAP at the endoplasmic reticulum (ER) through its N-terminal region [9,13], whereas binds the scramblase XK at the plasma membrane through the VAB domain [10], VPS13B interacts with Sec23 at the ERES [12], VPS13C binds Rab7 at the endo/lysosomes [7], and VPS13D binds the ESCRT-III component TSG101 [15]. Once lipids are delivered to the outer leaflet of the nascent membrane, certain scramblases transfer the lipids from the outer leaflet into the inner leaflet to facilitate the expansion of the membrane bilayers. Till now, several classes of scramblases have been identified at various membranes, including TMEM16, XK, TMEM41B, VMP1, ATG9, Class A GPCRs, and CLPTM1L. Among them, TMEM16, Class A GPCRs, and XK act as the scramblases in the plasma membrane to transfer lipids [10,16,17]. TMEM41B, VMP1, and CLPTM1L function in the ER [18], and ATG9 is recruited to the isolated membrane to fulfill the role of scramblase in the autophagy pathway [19].

We and other research teams have demonstrated that Golgi-mediated vesicular transport is crucial for the biogenesis of the IMC in *T. gondii* [20–24]. However, phospholipids are predominantly synthesized in the ER, Mitochondrion and Golgi Network of the parasite [25–33]. Hence, it is reasonable to speculate that a pathway exists in these parasites that directly mediates the transport of bulk lipids from the sites of lipid synthesis to the IMC and elsewhere. Furthermore, an efficient continuous supply of bulk lipids may be required for the membrane expansion of the IMC during daughter cell budding. A previous study has also indicated that MCSs exist at the IMC [34], but whether they contribute to the lipid transport to the IMC and mechanisms underlying the formation of flattened vesicles remain completely unknown. This study identified an ortholog of VPS13 in *T. gondii* (TgVPS13A), which interacts with a VAMP-associated protein (VAP) in the ER (TgVAP) and with a major facilitator superfamily (MFS) transporter located in the IMC (TgDAT1). TgVPS13A, TgVAP and TgDAT1 are required for the biogenesis of the IMC and progeny formation. The entire protein complex forms an inter-organellar bridge to mediate lipid transport at the ER-IMC interface.

## Results

### Identification of VPS13 and VAP family proteins in *T. gondii*

**Fig 1:**
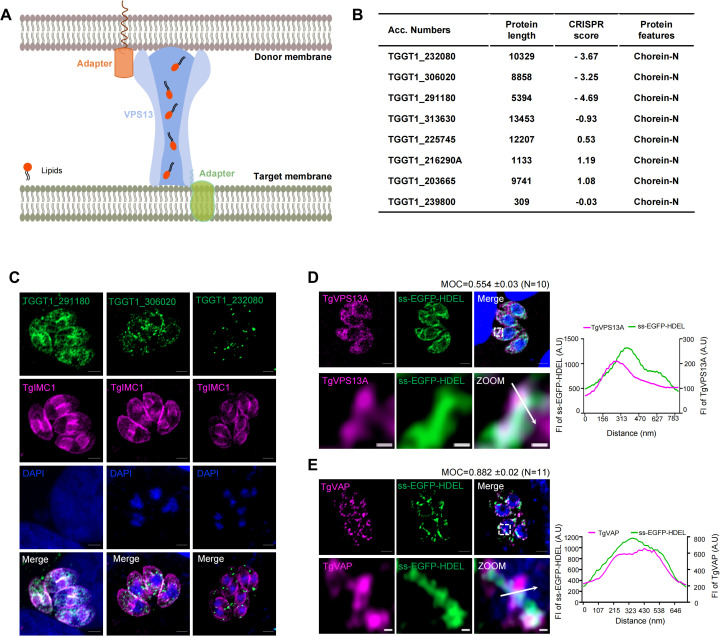
Identification of VPS13 and VAP family proteins in *T. gondii.* **(A)** A schematic diagram showing VPS13 family protein connecting donor and target organelles. **(B)** The list presents eight candidates identified by searching for the Chorein-N domain in the *T. gondii*. **(C)** IFA shows the localization of three of VPS13 proteins (CRISPR score < -1) in *T. gondii*. **(D)** IFA analysis showing TgVPS13A localization. The ER was labeled using a ss-EGFP-HDEL fusion protein (TgSAG1 signal peptide fused to EGFP-HDEL under the tubulin promoter). Colocalization of TgVPS13A with ss-EGFP-HDEL was quantified in ImageJ and is presented as mean ± SEM. Shown are Manders’ Overlap Coefficient (MOC) tM1 values for individual parasites. “N” indicates the number of parasites. (E) IFA showing colocalization of TgVAP with the ER marker ss-EGFP-HDEL. Quantification of TgVAP colocalization with ss-EGFP-HDEL is presented as mean ± SEM. Shown are tM1 values for individual parasites. “N” represents the number of parasites. Magenta: rabbit anti-HA antibodies; anti-TgIMC1 polyclonal antibodies; Green: Mouse anti-HA antibodies and EGFP signals; Blue: DAPI. Scale bars indicate 2 µm in the merged panels and 0.2 µm in the zoomed panels.

VPS13 family proteins function as lipid transporters by bridging membrane contact sites through interactions with adaptor proteins at donor and target organelles (Fig 1A). Despite their well-characterized roles in other organisms, their involvement in lipid transport to the IMC in *T. gondii* remains unknown. To address this question, we searched the ToxoDB database for proteins containing the Chorein-N domain (PF12624), which yielded eight candidates (Fig 1B). All of the candidates contained a clear Chorein-N domain as determined by the CD-Search tool, and three of these proteins had CRISPR scores lower than -1.0 (Fig 1B). Given the critical role of the IMC in parasite fitness, we focused on these three candidates. Further bioinformatic analysis indicated that these VPS13 orthologs harbor conserved domains characteristic of the VPS13 family, including a Chorein-N domain followed by an extended Chorein region and a VPS13 adaptor-binding region (VAB), and α-helical region (called the ATG2-C domain due to its homology with a corresponding region in ATG2) and Pleckstrin Homology (PH) domain at the C terminus. We investigated the subcellular localization of the selected candidates by generating HA-tagged parasite strains. Immunofluorescence analysis revealed that all three proteins displayed endomembranous or punctate localization in the parasites. Notably, TGGT1_291180 (TgVPS13A) also accumulated at the IMC buds, suggesting its specific involvement in IMC biogenesis during daughter budding (Fig 1C). Our structure modeling of TgVPS13A revealed a bridge-like central domain flanked by the N-terminal chorein and C-terminal VAB and ATG2-C domains, positioned for interaction with partner proteins in the donor and acceptor membranes (S1A Fig). The endoplasmic reticulum (ER) is the primary site where most phospholipid synthesis occurs. Next, we determined to detect the association of the TgVPS13A to the ER, we inserted an SmFP-HA tag at the C-terminal through the CRISPR/Cas9 method (S1B Fig). The integration of the tags was confirmed by diagnostic PCR (S1C Fig). The results indicated that partial signal of TgVPS13A-SmFP-HA merged with that of the ER marker ss-EGFP-HDEL (Fig 1D).

In mammalian cells, VPS13 anchors to the ER by binding integral membrane proteins known as VAMP-associated proteins (VAPs) through its N-terminal region, thereby facilitating lipid transport at MCSs [8,12]. Through a blast search using human VAPA and VAPB as baits, we found a unique VAP protein in *T. gondii* (TgVAP). TgVAP is phylogenetically conserved across alveolates, forming a distinct cluster separated from its human and yeast orthologs (S2A Fig). Sequence alignment of TgVAP with *Hs*VAP1/2 and *in silico* modeling displayed the occurrence of a conserved MSP domain for interaction with the FFAT motif of TgVPS13A (S2B Fig). Alphafold models of TgVPS13A N-terminus and TgVAP have been docked together using HADDOCK. The predicted complex reiterated the strong possibility of TgVAP binding to TgVPS13A N-terminus as these models ranked higher in the cluster with the best scores (S2C Fig). We inserted a 6 × HA tag at the N- terminal through the CRISPR/Cas9 method. Through an IFA analysis, we found 6 × HA-TgVAP localized primarily in the ER (Fig 1E). The ER localization of TgVPS13A and TgVAP proteins is consistent with the hyperLOPIT database [35]. Collectively, our data suggest the presence of a lipid transport complex formed by TgVPS13A and TgVAP between the parasite ER and IMC.

### TgVPS13A is partially associated with the nascent IMC

**Fig 2:**
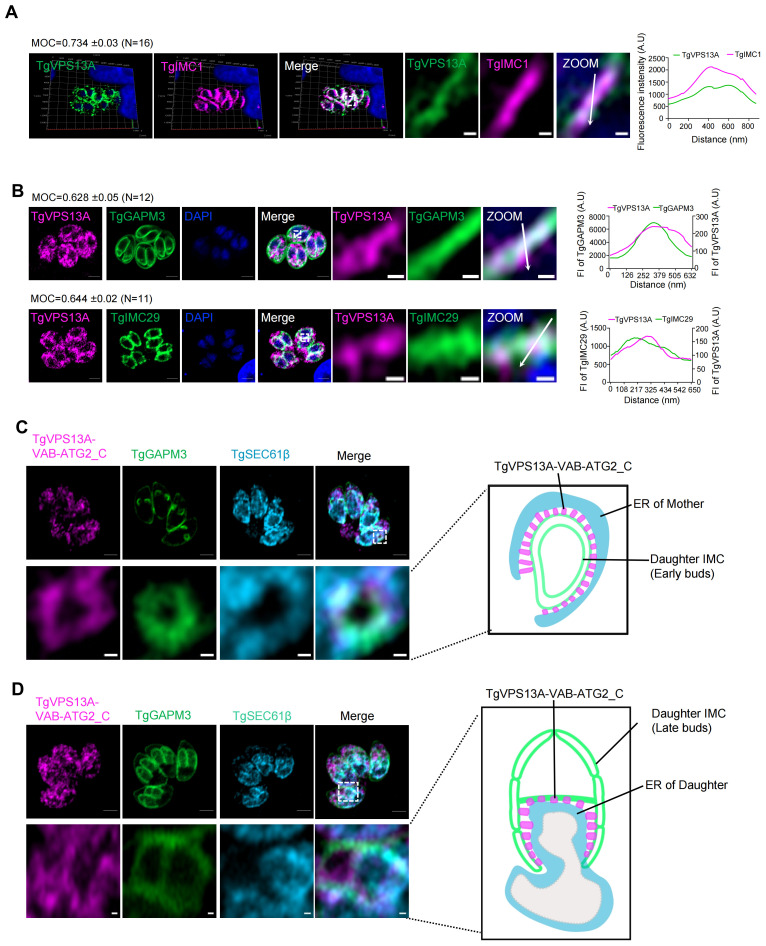
TgVPS13A is partially associated with the nascent IMC. **(A)** IFA showing 3D colocalization. The signal intensity of TgVPS13A-SmFP-HA across the TgIMC1 distribution is graphed. Manders’ Overlap Coefficient (MOC) was quantified in ImageJ and is presented as mean ± SEM. Shown is Manders’ tM2 (above TgIMC1 autothreshold). “N” indicates the number of parasites. **(B)** Subcellular localization of TgVPS13A-SmFP-HA assessed by co-staining with TgGAPM3 and TgIMC29. Parasites express C-terminally EGFP-tagged TgIMC29 and TgGAPM3. MOC was quantified in ImageJ and is presented as mean ± SEM. Shown is Manders’ tM1 (above TgIMC29/TgGAPM3 autothreshold). “N” represents the number of parasites. **(C** and **D)** IFA showing the colocalization of TgVPS13A-VAB-ATG2-C with TgSEC61β and TgGAPM3. Magenta: anti-TgIMC1 polyclonal antibodies, rabbit anti-HA antibodies, mouse anti-Myc antibodies; Green: rabbit anti-HA antibodies and EGFP signals; Blue: DAPI; Cyan: rabbit anti-V5 antibodies. Scale bars indicate 2 µm in the merged panels and 0.2 µm in the zoomed panels.

To ascertain the association of TgVPS13A with the IMC, we conducted its 3D colocalization analysis with TgIMC1. The fluorescence signal of the two proteins matches constantly over the distance at different stages of parasite development (Fig 2A). To monitor the dynamics of the TgVPS13A signal during daughter parasite emergence, we inserted a GFP tag at the C-terminus of TgGAPM3 or TgIMC29 to mark the nascent progeny. Subsequently, the colocalization of TgVPS13A with TgIMC29 or TgGAPM3 was assessed by IFA. TgVPS13A was indeed associated with the daughter cells during the budding (Fig 2B). In principle, both the mother and daughter ER could contribute the lipids needed for the budding IMC. We therefore examined the localization of TgVPS13A at the interface between the budding IMC and the ER of the mother or daughter cells. To investigate this, we inserted an EGFP or a 3xV5 tag at the C-terminus of TgGAPM3 (budding IMC) and TgSEC61β (ER) (Fig 2C-2D). TgSEC61β is an ortholog of human SEC61β, a commonly used ER marker [7]. We confirmed its localization using a known ER marker in *T. gondii* (S2D Fig). TgVPS13A was detectable at the interfaces of the budding IMC with both the mother and daughter ER.

Our findings also demonstrated that the VAB domain, in conjunction with the ATG2-C region of TgVPS13A, is sufficient for its localization at the IMC (Fig 2C-2D). Consequently, stable transgenic parasites expressing SmFP-MYC-tagged VAB-ATG2-C region of TgVPS13A were selected to examine the TgVPS13A expression at the interface between the ER and the IMC. The results indicated that the ER of the mother parasites interacts with the emerging buds of daughter IMCs during the initial phase (Fig 2C). As the progeny develop, the ER of the budding parasites starts associating with the middle region of the nascent IMC, but not the cap (Fig 2D). It has been reported that the cap region develops distinctly compared to the bulk IMC [36], which is consistent with our datasets.

### TgVPS13A is essential for parasite survival and the IMC assembly

**Fig 3:**
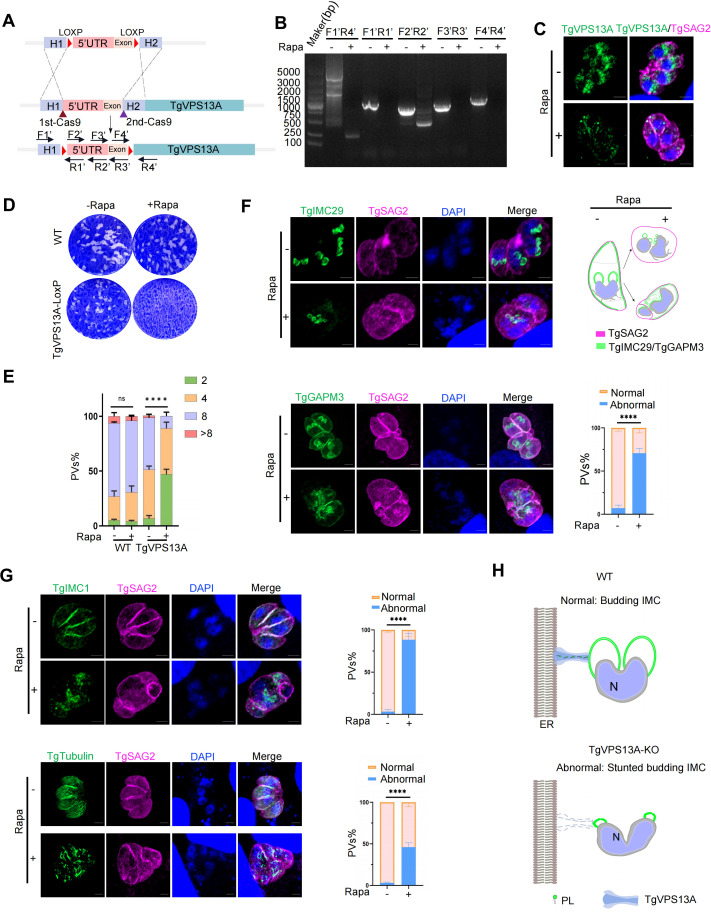
TgVPS13A is essential for parasite survival and is necessary for the assembly of the IMC. **(A)** The schematic representation illustrating the DiCre-LoxP system used to conditionally deplete TgVPS13A. **(B)** PCR analysis confirming the N-terminal recombination of TgVPS13A involving the two LoxP sites. F1’/R4’(full-length), F1’/R1,’ F2’/R2,’ F3’/R3,’ and F4’/R4’ amplify fragments of 3781 bp, 946 bp, 820 bp, 917 bp, and 1158 bp, respectively. Upon 96h of Rapa treatment, F1’/R4’ (full-length) amplify a 167 bp fragment; F1’/R1,’ F3’/R3,’ and F4’/R4’ do not amplify any fragments; and F2’/R2’ amplify non-specific fragments. **(C)** IFA analysis showing the expression of TgVPS13A in parasites cultured with or without Rapa for 96 h. Parasites were pretreated with Rapa for 72 h, and egressed parasites were subsequently inoculated into fresh BJ-5ta cell monolayers and cultured for an additional 24 h in the presence of Rapa, resulting in a total treatment duration of 96 h. **(D)** Plaque assays were conducted by infecting BJ-5ta cells with the specified parasites for 9 days, either in the presence or absence of Rapa. **(E)** Quantification of the replication of the indicated strains grown in BJ-5ta cells for 96 h in the presence or absence of Rapa. Parasites were pretreated with Rapa for 72 h, and egressed parasites were subsequently inoculated into fresh BJ-5ta cell monolayers and cultured for an additional 24 h in the presence of Rapa, resulting in a total treatment duration of 96 h. Data represents the mean  ±  SD from three independent experiments, analyzed using two-way ANOVA, with Tukey multiple comparison test, ****P < 0.0001. **(F)** IFA analysis of IMC biogenesis involved staining for TgIMC29 and TgGAPM3. The abnormal and normal PVs were counted from TgGAPM3. Data are represented as mean ± SEM from three independent slides, and at least 100 PVs were counted per slide. The difference in the number of abnormal PVs was statistically analyzed by Unpaired t-test; ****P < 0.0001. Schematic diagram of the assembly of early buds of daughter IMCs in TgVPS13A -deficient parasites. **(G)** IFA analysis of the IMC-associated cytoskeleton by examining the localization of TgIMC1 and TgTubulin in TgVPS13A-deficient parasites. The abnormal and normal PVs were counted from G. Data are mean ± SEM from three independent slides, and at least 100 PVs were counted per slide. The difference in the number of abnormal PVs was statistically analyzed by Unpaired t-test; ****P < 0.0001. Green: rabbit anti-HA antibody, rabbit anti-TgIMC1 polyclonal antibodies, rabbit anti-TgTubulin polyclonal antibodies, and EGFP signal; Magenta: rabbit and mouse anti-TgSAG2 polyclonal antibodies; Blue: DAPI. Scale bars represent 2 µm. **(H)** A schematic model of the IMC biogenesis and daughter cell budding in TgVPS13A-delepleted parasites. PL indicates Phospholipid. N’ indicates the nucleus.

To investigate the physiological role of TgVPS13A in IMC biogenesis and parasite survival, we first attempted to regulate its expression by the AID and TATi systems [37,38]. However, these efforts were unsuccessful. Thus, we adapted the DiCre system for a conditional depletion of TgVPS13A [39]. We inserted two loxP sites flanking the promoter region and the third exon of TgVPS13A in parasites expressing a rapamycin-inducible DiCre recombinase (Fig 3A). The correct insertion of the sequences was verified through diagnostic PCRs and sequencing analysis (Fig 3B). The expression of TgVPS13A remained unaffected in routine parasite cultures (-rapamycin). Upon treatment with rapamycin, the promoter region and the 5’-fragment of the *TgVPS13A* gene flanked by the loxP sites could be efficiently excised from the genomic DNA, resulting in the blockage of protein expression (Fig 3C). A depletion of TgVPS13A expression was confirmed by IFA (Fig 3C). We further quantified the regulatory level of TgVPS13A by IFA, and found that the expression level of TgVPS13A gradually decreased upon treatment with Rapa (S2E Fig). Knockdown of TgVPS13A severely impaired the lytic cycle, indicated by a lack of plaque formation in host-cell monolayers following rapamycin treatment. The presence of occasional tiny plaques may be attributed to incomplete recombination by the DiCre system (Fig 3D). Intracellular replication of the rapamycin-treated parasites was also compromised (Fig 3E).

We subsequently investigated early bud assembly by staining for the daughter IMC proteins TgIMC29 and TgGAPM3 in TgVPS13A-deficient parasites. The absence of TgVPS13A led to defects in daughter bud development, including asynchronous budding and in some parasites, complete absence of bud formation (as evidenced by TgIMC29 staining), collapse of the nascent IMC, and morphological abnormalities (as revealed by TgGAPM3 staining) (Fig 3F). Immunostaining of the IMC-associated cytoskeleton (TgIMC1 and TgTubulin) revealed similar defects in IMC organization and failure of subpellicular microtubules (SPMTs) assembly (Fig 3G). Taken together (schematized in Fig 3H), our data show that TgVPS13A is required for IMC biogenesis and budding in *T. gondii*.

### TgVAP in the ER is required for the parasite survival and IMC biogenesis

**Fig 4:**
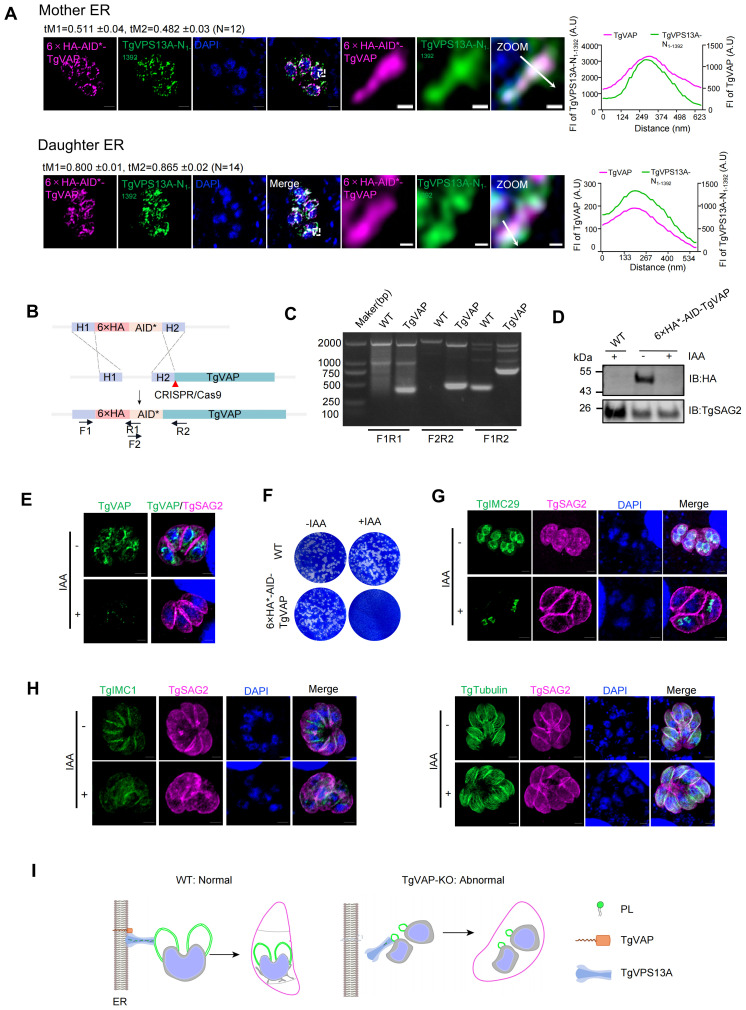
TgVPS13A is anchored to the ER by binding to TgVAP. **(A)** IFA was performed to examine the colocalization of TgVAP with TgVPS13A-N1-1392aa before and after division. Parasites were transfected with a plasmid expressing TgVPS13A-N1-1392aa-SmFP-Myc under the native promoter. MOC was quantified in ImageJ and is presented as mean ± SEM. Shown are Manders’ tM1 (above TgVAP autothreshold) and tM2 (above TgVPS13A-N1-1392aa autothreshold). “N” represents the number of parasites. **(B)** A schematic diagram of inserting a 6 × HA-AID* tag at the N-terminal of TgVAP. **(C)** PCR identification of the 6 × HA-AID* tag inserted at the N-terminal of TgVAP. F1/R1, F2/R2, and F1/R2 (full-length) amplifies fragments of 366 bp, 395 bp, and 742 bp, respectively; in contrast, only F1R2 amplified a 379 bp fragment in the wild-type strain. **(D)** The expression of TgVAP was examined using western blot analysis. The parasites were cultured in the presence of IAA for 24 h in BJ-5ta cells and then subjected to the analysis. WT refers to the wild type. TgVAP expression was detected using anti-HA antibodies. **(E)** IFA analysis of TgVAP expression in parasites with and without IAA treatment for 24 h. **(F)** Plaque assays were conducted by infecting BJ-5ta cells with the parasites for 9 days, both in the presence and absence of IAA. **(G)** The parasites were cultured with or without IAA for 48 h to assess the biogenesis of the IMC in daughter cells, using TgIMC29 for staining. The EGFP tag was inserted at the C-terminus of TgIMC29 in the parasites. **(H)** IFA analysis showing the impact of TgIMC1 and TgTubulin in TgVAP-deficient parasites with and without IAA treatment for 48 h. **(I)** A schematic diagram of the effect of TgVAP on IMC biogenesis. Magenta: rabbit anti-HA antibody, mouse and rabbit anti-TgSAG2 polyclonal antibodies; Green: rabbit anti-HA antibody, mouse anti-Myc antibody, rabbit anti-TgTubulin polyclonal antibodies, EGFP signal and anti-TgIMC1 polyclonal antibodies. Scale bars measure 2 μm in the merged panels and 0.2 μm in the zoomed-in panels.

As described, we found a VAP ortholog, located in the parasite ER. TgVAP also partially co-localized with TgVPS13A at the IMC both in mature and daughter parasites (Fig 4A). To investigate the role of TgVAP in the survival and assembly of the IMC in budding progeny, we inserted a 6xHA-AID* tag at the N-terminal of TgVAP, allowing its regulation by 3-indole acetic acid (IAA). The 5’-genomic integration of the tag was verified by diagnostic PCR (Fig 4B-4C). Treatment with IAA induced efficient degradation of 6xHA-AID*-TgVAP in the parasites (Fig 4D-4E and S2F Fig). TgVAP-depleted parasites were compromised in their growth, as demonstrated by the lack of plaque formation in IAA-treated parasites cultured on host-cell monolayers (Fig 4F). The abnormal assembly of the IMC in the parasites was observed again when TgVAP was depleted, as evidenced by TgIMC29 and TgIMC1 staining. As evidenced by TgIMC29 staining, stunted bud growth was observed again in the TgVAP-depleted parasites phenocopying TgVPS13A depletion (Fig 4G). Immunostaining of TgIMC1 revealed morphological abnormalities of IMC as well (Fig 4H). However, no obvious change was observed in the SPTMs assembly upon knockdown of TgVAP (Fig 4H). In brief, TgVAP anchored in the parasite ER is needed for the IMC biogenesis and budding (Fig 4I).

### TgDAT1 is a physiologically-essential IMC-localized lipid scramblase

**Fig 5:**
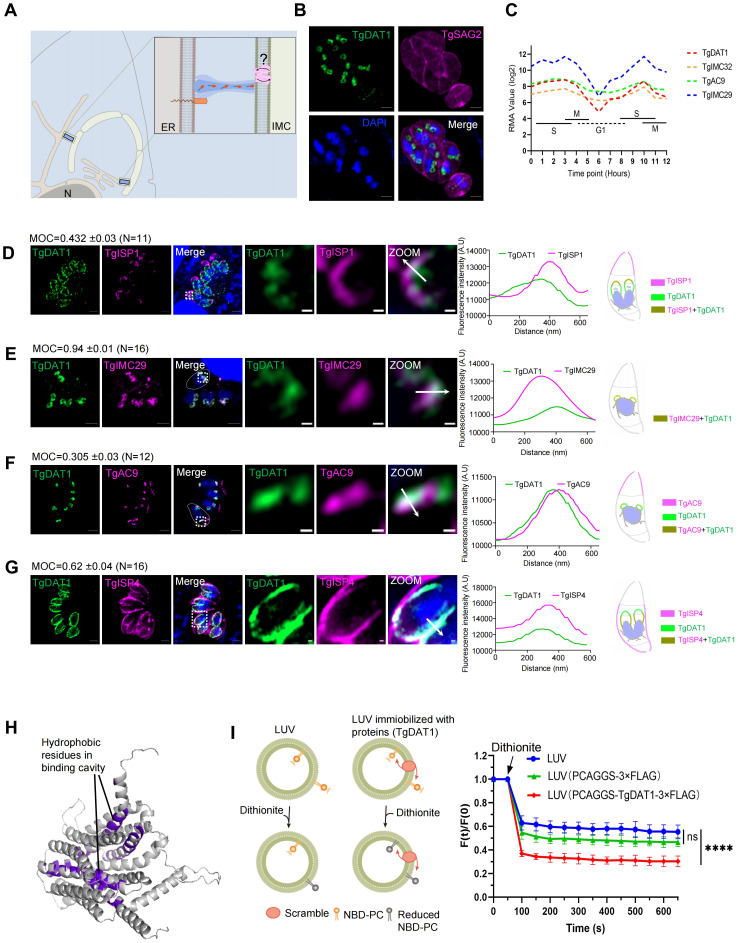
TgDAT1 is a physiologically-essential IMC-localized lipid scramblase. **(A)** A hypothetical model for Vps13-mediated lipid trafficking to IMCs involving a scramblase. **(B)** IFA showing the localization of TgDAT1. This strain was characterized by an endogenous insertion of a 3 × HA tag at the C-terminus of TgDAT1. **(C)** Robust Multi-array Average (RMA) analysis of the expression of the indicated genes is presented across the cell cycle. **(D-G)** IFA analysis of TgDAT1 subcellular localization co-stained with TgIMC29, TgAC9, TgISP1, and TgISP4. MOC was quantified in ImageJ and is presented as mean ± SEM. Shown is Manders’ tM2 (above autothreshold of TgISP1/TgIMC29/TgAC9/TgISP4). “N” represents the number of parasites. Green: rabbit anti-HA; Magenta: mouse anti-MYC and mouse anti-TgSAG2 polyclonal antibodies. Scale bars: 2 μm (merged panels) and 0.2 μm (zoomed panels). **(H)** Surface models of TgDAT1 predicted by Alphafold 3, and the PYMOL was used to highlight the hydrophobic residues in binding cavity (purple). **(I)** A schematic diagram of the dithionite assay. Examination of the scramblase activity of TgDAT1 was conducted through the dithionite assay, with LUV and PCAGGS-3 × FLAG serving as a control. Data are presented as the fluorescence intensity (FI) at indicated time points, normalized to the initial FI. Data represents the mean  ±  SD from three independent experiments. The data were statistically analyzed the Wilcoxon Signed Rank Test; ****P < 0.0001. LUV represents large unilamellar vesicles without protein.

The requirement for TgVPS13A-VAP proteins in nascent alveolar biogenesis implies the presence of a lipid scramblase in the IMC vesicle membrane. Following lipid delivery to the outer leaflet of the nascent IMC, scramblases mediate lipid transfer from the outer to the inner leaflet, facilitating expansion of the membrane bilayer (Fig 5A). Therefore, we performed a global screening by endogenously inserting a hemagglutinin (HA) tag using the CRISPR/Cas9 method (S3A Fig). We included the annotated transporters with more than two transmembrane domains from ToxoDB in this screening, excluding those with a CRISPR score greater than -1.0. In this screening, we detected fluorescence signals from 64 out of 71 annotated putative transporters encoded in the genome of *T. gondii* (S1 Table and S3B Fig). Our search identified TGGT1_258700 belonging to the major facilitator superfamily (MFS) of transporters, similar to other SPNS transporters [40,41], resulting in localization of the fusion protein in the IMC (Fig 5B). The RNA expression profile of this transporter (termed as ***D***aughter ***A***lveoli ***T***ransporter 1, DAT1) is similar to AC9, IMC29, and IMC32 (Fig 5C). An examination of the cellular localization of the transporter, through co-staining with the ISPs, revealed that this protein was predominantly expressed in early daughter buds but was rarely found in mature parasites (Fig 5D). Additionally, further co-localization analysis with the protein markers of different IMC regions revealed that its localization at the IMC during emerging process of daughter buds (Fig 5E-5G). The Alphafold structure of TgDAT1 showed that its binding cavity is rich in hydrophobic residues, containing only a few charged residues (10/124) (Fig 5H). This suggests that its substrate is likely lipophilic in nature. We then investigated whether TgDAT1 can transport lipids from the inner to the outer leaflet of liposomes using the previously established dithionite assay [19,42]. In this assay, liposomes containing fluorescent 7-nitro-2, 1, 3-benzoxadiazol (NBD) acyl-labeled phospholipids are treated with dithionite, a membrane-impermeable reducing agent. Dithionite reduces and irreversibly quenches the fluorescence of NBD-PC located in the outer leaflet of the liposome (Fig 5I). TgDAT1 was expressed in HEK293T cells and purified [43] for this experiment (S4A Fig). After detecting the fluorescence of NBD-PC for 50 seconds, dithionite was added, and measurements continued for an additional 600 seconds. The results showed that the fluorescence curve of TgDAT1 significantly dropped compared to the controls (Fig 5I), indicating that TgDAT1 acted as a scramblase.

### TgDAT1 plays a vital role in biogenesis of budding IMC

**Fig 6:**
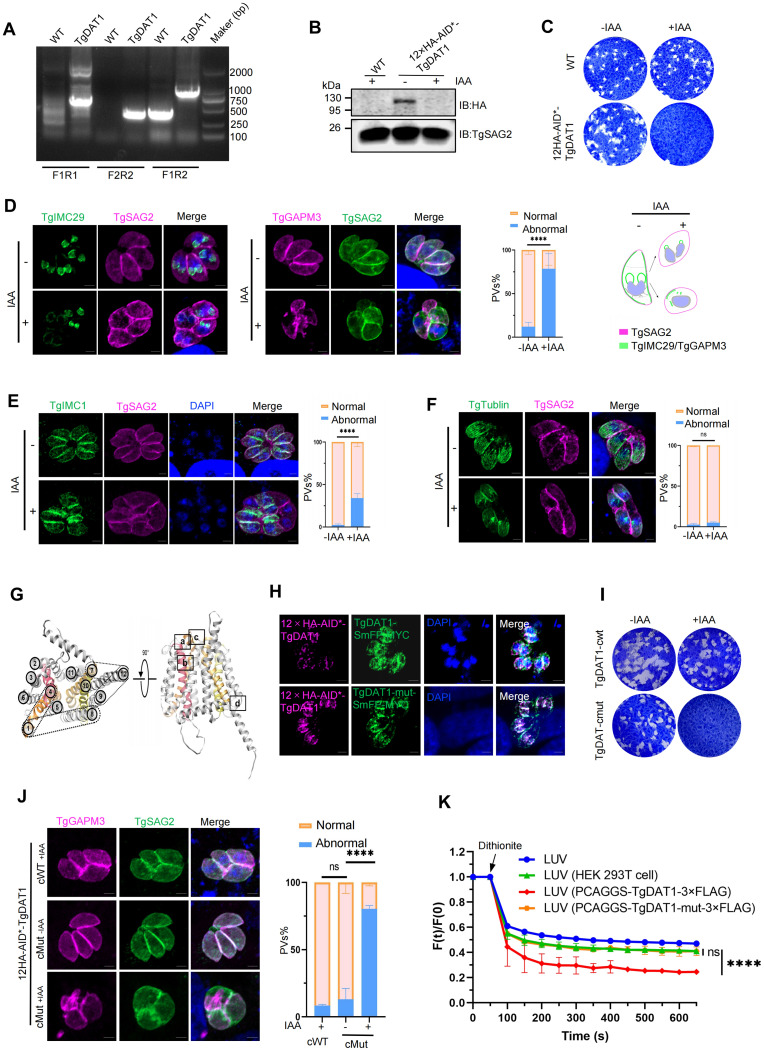
TgDAT1 plays a vital role in biogenesis of budding IMC. **(A)** PCR identification of the 12 × HA-AID* tag inserted at the N-terminal of TgDAT1. F1/R1, F2/R2, and F1/R2 (full-length) amplifies fragments of 635 bp, 365 bp, and 964 bp, respectively; whereas for the wild-type strain, only F1/R2 amplifies a fragment of 409 bp. **(B)** Expression of TgDAT1 was examined using western blot analysis. The parasites were cultured in the presence of IAA for 24 h in BJ-5ta cells and then subjected to the analysis. WT refers to the wild type. TgDAT1 expression was detected using anti-HA antibodies. **(C)** Plaque assays were performed by infecting BJ-5ta cells with the parasites for 9 days in the presence or absence of IAA. **(D)** The biogenesis of the daughter IMC was investigated by examining the localization of TgIMC29 and TgGAPM3 in TgDAT1-deficient parasites. An EGFP tag or a mCherry tag was inserted at the C-terminal of TgIMC29 or TgGAPM3 in the 12 × HA-AID*-TgDAT1 cell line respectively. Parasites were treated with IAA for 48 h. The abnormal and normal PVs were counted from TgGAPM3. Data are mean ± SEM from three independent slides, and at least 100 PVs were counted per slide. The difference in the number of abnormal PVs was statistically analyzed by Unpaired t-test; ****P < 0.0001. Schematic diagram of the assembly of early buds of daughter IMCs in the TgDAT1-KO parasites. **(E)** The biogenesis of the IMC was investigated by staining with IMC1 antibodies in TgDAT1-deficient parasites. The parasite lines were cultured with or without IAA for 48 h and stained with antibodies against TgSAG2 and TgIMC1. Data are mean ± SEM from three independent slides, and at least 100 PVs were counted per slide. The difference in the number of abnormal PVs was statistically analyzed by Unpaired t-test; ****P < 0.0001. **(F)** IFA analysis showing the assembly of TgTubulin in TgDAT1-deficient parasites. The abnormal and normal PVs were counted. Data are mean ± SEM from three independent slides, and at least 100 PVs were counted per slide. The difference in the number of abnormal PVs was statistically analyzed by Unpaired t-test. **(G)** A model of TgDAT1 and the potential sites of salt bridges predicted by AlphaFold and Chimera. **(H)** IFA analysis showing the localization of TgDAT1 mutants at the IMC. Parasites were transfected with plasmids expressing mutated versions of TgDAT1-SmFP-MYC under the control of the native promoter, and those stably expressing these mutants were selected using pyrimethamine. **(I)** Plaque assays were performed by infecting BJ-5ta cells with TgDAT1 mutants and wild-type parasites for 8 days, both in the presence and absence of IAA. **(J)** IFA showing the localization of TgGAPM3 in salt-bridge mutants of TgDAT1. An endogenous mCherry tag was inserted into the C-terminus of TgGAPM3 in parasites. The abnormal and normal PVs were counted. Data are mean ± SEM from three independent slides, and at least 100 PVs were counted per slide. The difference in the number of abnormal PVs was statistically analyzed by Unpaired t-test; ****P < 0.0001. Magenta: rabbit anti-TgSAG2 polyclonal antibodies, mCherry, rabbit anti-HA; Green: EGFP signal, rabbit anti-TgSAG2, rabbit anti-TgTubulin polyclonal antibodies, and mouse anti-MYC; Blue: DAPI. Scale bars: 2 μm. **(K)** Examination of the scramblase activity of TgDAT1 salt bridge mutant (TgDAT1-mut) was conducted through the dithionite assay. LUV: large unilamellar vesicles only; LUV (HEK293T cell): LUV immobilized with the elutes from the HEK293T cells; LUV (PCAGGS-TgDAT1–3 × FLAG): LUV immobilized with the elutes from the HEK293T cells transfected with PCAGGS-TgDAT1–3 × FLAG. Data are presented as the fluorescence intensity (FI) at indicated time points, normalized to the initial FI. Data represents the mean  ±  SD from three independent experiments. The data were statistically analyzed using the Wilcoxon Signed Rank Test; ****P < 0.0001.

To assess the physiological roles of TgDAT1 in *T. gondii*, we endogenously tagged a 12 × HA-AID* at the N-terminal of TgDAT1 (S4B Fig) and confirmed the correct integration of the tag through diagnostic PCRs and sequencing analysis (Fig 6A). The addition of 3-indoleacetic acid (IAA) resulted in efficient degradation of the protein, as indicated by IFA (S4C Fig) and western blotting (Figs 6B, S4D). We found that depletion of TgDAT1 inhibited the lytic cycle of parasites, as evidenced by the absence of plaque formation in IAA-treated parasites within HFF monolayers (Fig 6C).

Next, we investigated whether the IMC assembly was affected by the depletion of TgDAT1 by staining the nascently-recruited IMC proteins. Again, the absence of TgDAT1 resulted in stunted development of the IMC in daughter cells visualized by TgIMC29 or TgGAPM3 (Fig 6D), which resembled the TgVPS13A or TgVAP-deficient parasites. Although the IMC1 scaffold assembles in the absence of functional TgDAT1, its precise organization is perturbed, as reflected by the observed morphological changes. This phenotype phenocopies the IMC1 staining pattern observed in TgVAP-depleted parasites (Fig 6E). Our findings also revealed that the assembly of the SPMTs was not affected significantly (Fig 6F). Since the apical cap may form *via* a different mechanism, we stained AC9 (a cap alveolin) and found its localization was disrupted (S4E Fig). To further determine whether the growth-stunted daughter buds in the TgDAT1-depleted parasite comprise the cap region, we colocalized TgIMC29 with AC9. While the AC9 localization pattern was primarily disrupted, its staining was still visible in the growth-stunted buds. The signal for AC9 marked only the top region of the stunted buds but did not completely merge with TgIMC29 (S4F Fig).

The conformational dynamics of transporters, including the inward-facing (IF) and outward-facing (OF) transitions, are crucial for their substrate transport functions [44]. In MFS family proteins, these states may be stabilized by the formation and breaking of inter- and intra-domain salt bridges [41]. The predicted structure of TgDAT1 (Fig 6G) revealed 8 residues forming salt bridges. We mutated 7 residues (316D/N, 326E/Q, 397R/Q, 576K/C, 579K/C, 617R/Q, 722E/Q) and expressed the eventual TgDAT1 mutant in the 12xHA-AID*-TgDAT1 strain. Immunostaining showed that the TgDAT1 mutant could still be positioned in the IMC (Fig 6H), but it failed to rescue the parasite growth (Fig 6I) and IMC assembly in TgDAT1-depleted strain (Fig 6J). The results of the dithionite assay also further demonstrated that the mutation of the salt bridge lost its activity in flipping the NBD-PC (Fig 6K). Collectively, we show that TgDAT1 is an early-recruited scramblase at IMC that is required for the daughter IMC assembly.

### TgDAT1 is required for the phospholipid targeting to and building of the alveoli

**Fig 7:**
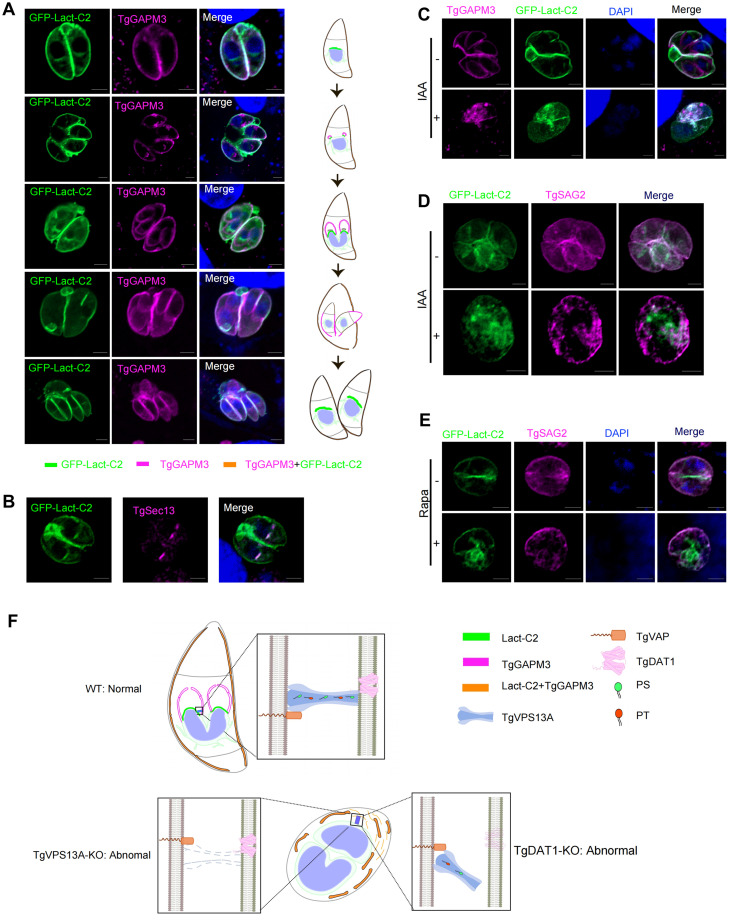
TgDAT1 is required for the phospholipid targeting to and building of the alveoli. **(A)** IFA showing colocalization of Lact-C2 and TgGAPM3 in IMCs during the complete Toxoplasma cycle. The TgDAT1-deficient strain with the mCherry inserted into the C-terminus of TgGAPM3 was transfected with a plasmid expressing GFP-Lact-C2, which is expressed under the GRA1 promoter, and those stably expressing strains were selected using pyrimethamine. **(B)** IFA analysis showing colocalization of Lact-C2 and the ER arrival sites (TgSec13) in *T. gondii*. Endogenous insertion of a 3 × V5 tag into the C-terminus of TgSec13 in parasites stably expressing GFP-Lact-C2. **(C** and **D)** IFA analysis showing colocalization of LACT-C2 and TgGAPM3 in TgDAT1-deficient parasites. **(E)** IFA analysis revealing the localization of GFP-Lact-C2 in TgVPS13A-deficient parasites. Magenta: rabbit anti-TgSAG2 polyclonal antibodies, mCherry signal and mouse anti-V5. Green: EGFP signal; Blue: DAPI. Scale bars: 2 μm. **(F)** A schematic representation of PS/PT trafficking in TgDAT1- and TgVPS13A-deficient parasites.

In final assays, we examined whether membrane lipid bilayer is indeed affected upon protein depletion, leading to disrupted IMC assembly. We expressed GFP-Lact-C2 in the TgDAT1 and TgVPS13A mutants. GFP-Lact-C2 is a biosensor of phosphatidylserine (PtdSer) [45] and has been adopted in *T. gondii* tachyzoites to monitor the subcellular localization of PtdSer and its naturally abundant structural homolog phosphatidylthreonine (PtdThr) [46,47]. We tested localization dynamics of GFP-Lact-C2 and found that it was primarily present in the IMC of TgGAPM3-stained mature parasites, but not in the early progeny (Fig 7A). The biosensor also partially accumulated at the ER arriving sites (Fig 7B). Upon TgDAT1 depletion, GFP-Lact-C2 signal collapsed similar to that of TgGAPM3 (Fig 7C-7D). We also examined its localization in the TgVPS13A-deficient parasite. Again, it was evidently mis-located and failed to reflect normal IMC (Fig 7E). The data infer a role of TgDAT1 and TgVPS13A in normal lipid expression in the alveoli membrane (Fig 7F).

## Discussion

Inner membrane complex is a defining feature of alveolate organisms. Although the proximity -dependent biotinylation approaches have identified numerous novel IMC-associated proteins—including alveolins [48–51], IMC Sub-compartment Proteins (ISPs) [52], ISCs and TSCs [2], the mechanisms underlying IMC biogenesis, particularly the formation of the alveolar membranes, remain poorly understood. Gliding-associated proteins (GAPs) are also implicated in IMC assembly, yet their precise roles and mechanisms of action remain elusive. Previous studies indicate that vesicle trafficking via the Golgi apparatus is essential for IMC biogenesis. For example, Brefeldin A treatment disrupts the IMC formation [20,21]. Furthermore, key components of the vesicular transport machinery, such as TgVPS45 (an SM protein), TgStx6 (a Qc SNARE) [53], and Rab11A (a GTPase) [53], have been shown to be required for proper IMC development. In this study, using the well-established alveolate model parasite *T. gondii*, we found a novel bridge-like TgVPS13A plays a critical role in IMC biogenesis. Given the conserved function of VPS13 family proteins in mediating intermembrane lipid transfer across eukaryotes, the TgVPS13A is likely involved in lipid transport from the ER to the IMC. Our discovery of this previously uncharacterized protein machinery reveals a new mechanism for inter-organelle lipid trafficking, offering important insights into membrane and organelle biogenesis in *T. gondii* and related alveolates, including clinically significant protozoan parasites.

As mentioned above, our initial exploration of VPS13 orthologues was conducted by searching for candidates containing the Chorein-N domain, which led to the identification of eight candidates (Fig 1B). Further BLAST analysis against the UniProt database, using yeast and the four human VPS13 orthologs (S5A Fig) as queries, identified three additional candidates, including TGGT1_210700, TGGT1_248510 and TGGT1_216335 (S5B Fig). Maximum Likelihood (S6A Fig) and Neighbor-Joining (S6B Fig) analyses of full-length VPS13 sequences established a robust topology, clearly separating Apicomplexan, Metazoan, and Fungal lineages with high bootstrap support. Notably, TgVPS13A (TGGT1_291180) exhibits accelerated sequence divergence, suggesting adaptation to its unique role in the IMC while retaining the ancestral core machinery. A parallel pattern of paralogous divergence is evident in *Plasmodium* (PF3D7_1346400) and *Cryptosporidium* (GY17_00001497) orthologs which form a deeply conserved clade distinct from other VPS13 paralogs. This topology supports a model of subfunctionalization, where VPS13 paralogs preserve conserved lipid-transport machinery while diverging to meet lineage-specific constraints. This hypothesis is reinforced by N-terminal domain phylogeny (S7 Fig), which clusters these three orthologs together. Given the N-terminus mediates membrane recognition and lipid transfer, this tight clustering indicates that despite vast evolutionary distances, the fundamental role of these proteins in maintaining membrane homeostasis remains strictly conserved.

VPS13 family proteins interact with an adaptor in the targeting membrane via its C-terminal VAB domain, and binds with the ER-resident VAP through its N-terminal domain. We tested the interaction of TgVPS13A with TgDAT1 or TgVAP. TgVPS13A and TgDAT1 by using co-immunoprecipitation with proteins expressed in HEK293T cells and performed in silico docking of respective predicted structures with HADDOCK (S8A-S8D Fig). Our results showed that TgDAT1 and TgVAP could bind the VAB domain or N-terminal of TgVPS13A, suggesting TgVAP and TgDAT1 could serve as a receptor for TgVPS13A in the ER or IMC. Furthermore, our results demonstrate that TgVPS13A, TgVAP, and TgDAT1 co-localize within the daughter IMC, suggesting they exist as part of a protein complex (S8E-S8F Fig). However, further investigation is warranted to confirm whether these proteins form a complex bridging the ER and the IMC. In addition, the observed lipid transport defects and IMC biogenesis abnormalities upon protein depletion are consistent with disrupted ER-IMC MCS function. While ultrastructural analysis by electron microscopy is still required, these data support the existence of MCSs between nascent IMC buds and the ER of maternal or daughter parasites. VPS13 family proteins are known to serve as organelle tethers that facilitate membrane contact site (MCS) formation [8]. It will therefore be important to determine whether depletion of the TgVAP, TgVPS13A or TgDAT1 affects ER-IMC MCS architecture.

Accumulating evidence has demonstrated that the ER is morphologically and functionally heterogeneous, comprising distinct subdomains. Given that the ER communicates with various organelles through MCSs, it is plausible that these MCSs are not randomly distributed but are instead spatially organized at specific ER subdomains, likely defined by distinct protein and lipid compositions. Our IFA data reveal that TgVPS13A does not co-localize extensively with ER markers, but rather exhibits partial co-localization, suggesting that it localizes to specific subdomains or sites within the ER. On the other hand, TgVPS13A localizes to the maternal ER–daughter IMC interface during early budding and subsequently forms a bridge at the daughter ER–daughter IMC interface in later stages (Fig 2C and 2D). While these observations await confirmation by electron microscopy, the colocalization signals may correspond to different ER-IMC interfaces depending on the stage of budding. This is consistent with previous reports that the cap region develops distinctly from the bulk IMC [36].

TgVPS13A-deficient parasites exhibited defective tubulin assembly—phenotypes that were not observed upon depletion of TgVAP or TgDAT1. Our previous study revealed that SPMT assembly is also disrupted upon TgVps45 depletion, a phenotype coupled with the failure of IMC biogenesis [22]. TgVps45 is an SM family protein involved in vesicular trafficking. In the present study, we observed that both TgVps13A and TgDAT1 are recruited at the very early stages of IMC biogenesis. Notably, however, we did not detect Vps45 at these early IMC buds. Therefore, we propose that the disruption of SPMT assembly is a secondary consequence of the failed delivery of IMC-anchored membrane proteins (such as GAPMs) via TgVps45-mediated vesicular trafficking. In this scenario, the absence of GAPMs leads to SPMT instability [54]. In our model, TgVPS13A and TgDAT1 initiate IMC bud formation, and subsequently, IMC-anchored membrane proteins are delivered by Vps45-mediated vesicle trafficking to stabilize the SPMT. Alternatively, the prominent dispersed staining pattern of TgVPS13A suggests additional localization to other membranes in *T. gondii*. Therefore, it is reasonable to propose that TgVPS13A plays a broader role in *T. gondii* beyond the ER-IMC interface and the observed defect might be a secondary consequence. In the case of VAP proteins, they are well-established ER-localized tethers that interact with multiple organelles at MCSs. While they are expected to interact with various FFAT motif-containing proteins, they are generally distributed throughout the entire ER membrane rather than being restricted to specific MCSs or subdomains. Consequently, depletion of TgVAP may disrupt multiple cellular processes, which is a critical consideration when interpreting the phenotypes of TgVAP-deficient parasites.

From the perspective of phospholipid metabolism, various phospholipids can be salvaged and/or synthesized *de novo* in *T. gondii* [55,56]*.* PtdCho and PtdEtn, the most abundant phospholipids in *T. gondii* are synthesized in the ER [25,31,57]. PtdEtn in *T. gondii* can also be produced from PtdSer by TgPSD1mt in the mitochondria [26] and by TgPSD1pv in the parasitophorous vacuole [58]. PtdSer and PtdThr are generated in the ER [27,32], and PtdIns in the Golgi apparatus [30], while phosphatidylglycerol (PtdGro) is thought to be produced in the ER and mitochondrion [28,29]. Recent work of Konishi *et al*. employed quick-freeze, deep-etch replica immunolabeling combined with electron microscopy to map the distribution of PtdSer and PtdEtn within the membrane leaflets of tachyzoites [59]. Additionally, GFP-C2-Lact-detected phospholipids (i.e., PtdSer and PtdThr) are enriched in the IMC besides the ER [46,47]. Furthermore, previous studies have demonstrated that maternal IMCs are recycled for daughter cell assembly [21]. These major lipids must be transported from the multiple sites of de novo synthesis or salvage to the IMC to support organelle biogenesis. Previous studies in other models have shown that MCSs exist between almost all opposing membranes to facilitate lipid exchange among organelles [60–62]. Collectively, these observations suggest that, in addition to the ER-IMC pathway, multiple routes originating from the Golgi apparatus mitochondria or other compartments may contribute to lipid delivery to the IMC. Future studies are warranted to elucidate how these mechanisms are functionally integrated. In this context, other putative VPS13 genes have been identified in the *T. gondii* genome in this study. It remains to be determined whether additional VPS13 homologs and MCSs might also facilitate inter-organelle lipid transport.

In this study, we provide evidence that TgDAT1 functions as a phospholipid scramblase. This activity likely facilitates lipid transfer from the outer to the inner leaflet of the nascent IMC membrane, thereby promoting bilayer expansion. While scramblases typically randomize phospholipid distribution between leaflets, phospholipids in the IMC are known to be asymmetrically distributed, with both PtdSer and PtdEtn primarily localized to the luminal leaflet [58]. Consequently, the mechanisms governing the establishment or maintenance of this specific asymmetry in the presence of scramblase activity remain unclear.

## Materials and methods

### Cell and Parasite strains culture

The hTERT-immortalized HFF cell line BJ-5ta (ATCC CRL-4001) was grown in Dulbecco’s Modiﬁed Eagle’s Medium (DMEM, Sigma-Aldrich, D6429) supplemented with 10% fetal bovine serum (CLARK, FB25015), 20% (v/v) Medium 199 basic (gibco, C11150500BT), and 1% penicillin/streptavidin. *T. gondii* RHΔhxgprtΔku80TIR1-FLAG were cultivated in BJ-5ta cells.

### Construction of plasmids

All primers used in this study are listed in S2 Table. The guide RNA sequences specific for TgVPS13A (TGGT1_291180), TgVAP (TGGT1_318160),TgDAT1 (TGGT1_258700), TgIMC29 (TGGT1_243200), TgGAPM3 (TGGT1_271970), TgSec61β (TGGT1_211040) andTgSec13 (TGGT1_201700) are listed in S3 Table. These sequences were designed using EuPaGDT (http://grna.ctegd.uga.edu/) and inserted into the PmeI site of the pCD-Cas9 vector [62] to construct CRISPR/Cas9 plasmids. The pCD-Cas9 vector contains the Cas9 gene and the TgU6 promoter, which drives the expression of the guide RNA.

The fragments for TgGAPM3-BirA*-3HA, ss-EGFP-HDEL, TgISP4–3 × MYC, TgISP1–3 × MYC, TgAC9–3 × MYC, TgIMC29–3 × MYC, and GFP-Lact-C2 were introduced into the pBluescript-DHFR vector under the control of either the the native, TgGRA1 or Tgβ-tubulin promoter. To express TgVPS13A-VAB-ATG2-C, TgVPS13A-N1-1392 and TgDAT1 in *T. gondii*, we amplified the promoter sequences and fused them with the coding sequences, a SmFP-MYC tag, and then cloned them into the pBluescript vector containing a DHFR cassette. For the TgDAT1 mutant, acidic or basic amino acids D316, E326, R397, K576, K579, R617, and E722 were replaced with neutral amino acids using overlapping PCR, and this was subsequently cloned into the aforementioned vector. To express TgVAP, TgVPS13A-N929-1392, TgVPS13A-VAB, TgDAT1 and TgDAT1-△N-1–303 in HEK293T cells, the open reading frames of these genes were amplified, and then introduced into the PCAGGS-3 × HA/PCAGGS-3 × FLAG vector.

### Generation of transgenic *T. gondii* strains

To construct AID-inducible conditional knockout strains, the RHΔhxgprtΔku80TIR1–3 × FLAG strain was co-transfected with a linearized DNA fragment and the pCD-Cas9 plasmid to insert the 12 × HA-AID* tag at the endogenous genomic loci of TgVAP and TgDAT1 at the N-terminus. To create LoxP sites in the genome locus of TgVPS13A, we amplified the promoter region and a fragment of the N-terminal of TgVPS13A from the genomic DNA of the parasites. This amplification was flanked by two LoxP sites using PCR. The resulting construct was then used to transfect DiCre parasites with CRISPR/Cas9 plasmids to develop the conditional knockout strain. To endogenously insert tags into the C-terminus of TgVPS13A, TgIMC29, TgGAPM3, TgSec61β, and TgSec13, parasites were co-transfected with the sequences containing the SmFP-HA, EGFP and mCherry tags and pCD-Cas9 plasmid.

### Parasite lines, transfections and selection

To obtain stable transgenic parasites, 1 × 10^7^ of fresh RHΔhxgprtΔku80TIR1–3 × FLAG or Dicre strains were transfected with 30 μg plasmid and 6 μg linearized DNA fragment by electroporation using standard procedures (1.5 kV, 50Ω, 25 μF and 2 mm cuvette) with the Gene Pulser Xcell (BIO-RAD). These parasites were allowed to grow in Vero cells for 10–16 h and then sorted with flow cytometry (SONY-SH800S). The sorted cells were inoculated into 96-well plates with fibroblast cell monolayers. After 7–8 days, monoclonal was screened by PCR to confirm correct integration. To select the strain by pyrimethamine, 1 × 10^7^ parasites were transfected by electroporation with 20 μg of the related plasmids. After 16 h, the transfected parasites were selected using pyrimethamine over three passages.

### Reagents and antibodies

500 mM IAA (Aladdin, I101074) was prepared with absolute ethanol. The final concentration of IAA is 500 μM. Rapamycin (MCE, HY-10219) was prepared at 10 mM in DMSO. The final concentration of Rapamycin is 50 μM. Mouse and rabbit anti-TgSAG2 (1:1000), rabbit anti-IMC1 and -TgTubulin used in immunofluorescence analysis (IFA) and western blotting (WB), were prepared previously in our lab [22]. Tag antibodies were used at the following dilutions: mouse anti-HA mAb (Sigma-Aldrich, H9658): 1:2000 (WB), 1:1000 (IFA); mouse anti-FLAG mAb (Sigma-Aldrich, F3165): 1:2000 (WB), 1:1000 (IFA); rabbit anti-HA mAb (Cell Signaling Technology, 3724S): 1:2000 (WB), 1:1000 (IFA); mouse anti-MYC (Cell Signaling Technology, 2276S): 1:2000 (WB), 1:1000 (IFA); rabbit anti-V5 mAb(Sigma): 1:1000 (IFA). Alexa Fluor 488 secondary antibody (Invitrogen, A11001): 1:1000 (IFA); Alexa Fluor 594 secondary antibody (Invitrogen, A11037): 1:1000 (IFA); DyLight 800-labeled anti-mouse IgG (SeraCare Life Sciences, 5230–0415): 1:10000 (WB).

### Western blotting

For Western blot samples, parasites or cells was collected and incubated with RIPA buffer for 30 min on ice. Afterwards, samples were centrifuged for 30 min at 13,000 rpm at 4°C and the supernatant was mixed with SDS loading dye (Solarbio). The samples were loaded onto a 10% SDS-PAGE gel followed by transfer onto a PVDF membrane. The PVDF membrane containing the transferred proteins was blocked with 5% skimmed milk in 1 × Tris-buffered Saline (TBS) buffer containing 0.5% Tween-20 for 1 h at room temperature. The PVDF membrane was then incubated with 1:2000 dilution of rabbit anti-HA monoclonal antibody (Cell Signaling Technology, 3724S) and 1:1000 dilution of rabbit anti-TgSAG2 antibody in blocking buffer for overnight at 4°C followed by 5 × washes of 1 × TBST. The blots were then incubated with 1:10000 dilution of IRDyeLight 800CW goat anti-Rabbit/Mouse IgG (926–32210/926–32211) in blocking buffer for 1 h at room temperature followed by 5x washes of 1x TBST. The probed PVDF membranes were visualized using Odyssey CLX (Li-COR).

### Immunofluorescence assay (IFA)

BJ-5ta monolayers grown on coverslips (CITOGLAS) or φ20 mm tissue culture dishes (Biosharp, BS-20-GJM) were infected with tachyzoites and grown at 37°C. Cells were subsequently fixed with 4% paraformaldehyde (PFA) (Biosharp, BL539A) for 30 min, and permeabilized (0.3% Triton X-100/PBS) for 10 min, blocked (5% BSA/0.5% Tween-20/PBS) for 1h at room temperature, and probed with primary antibodies 2 h prior to washing (0.5%Tween-20/PBS) and incubated with secondary antibodies (Alexa 488- or Alexa 594-conjugated goat anti-mouse/rabbit) for 1 h at room temperature in the dark. Coverslips were mounted on slides (Sail brand, 7101) using Antifade Mountant with DAPI (Biosharp, BL739B). Image acquisition was performed using an LSM 980 confocal scanning microscope equipped with a 100 × /1.4 NA objective with pixel size, XY = 120 nm/pixel resolution; Z = 350nm/pixel resolution. Images were captured with a LSM 980 internal GaAsP-PMT detectors (Zeiss, Germany) and acquired using Zeiss ZEN Blue (version 3.2) software. All Images were processed and exported as 24-bit JPG files using ZEN Blue (version 3.2). For 3D images, maximum intensity projections were generated from Z-stacks (step size: 110 nm). Capture the positioning status of 14–24 optical slices in total and synthesize a 3D image Zeiss ZEN Blue (version 3.2). For visualization only, brightness and contrast were adjusted linearly in Zeiss ZEN Blue (version 3.2).

### Protein expression and purification

TgDAT1 was expressed in HEK293T cells for 48h. The cells were pelleted and either used immediately or stored at -20°C. For purification, the cell pellets were lysed in 10 mM HEPES PH7.4, 100 mM NaCl, 19.6mM DDM, 1x protease inhibitor cocktail using 400 µL per 10 cm dish of cells, and mixed overnight at 4 °C. Cell lysates were centrifuged at 12, 000 rpm for 40 min, and the supernatant was incubated with preequilibrated anti-FLAG M2 affinity resin (Sigma-Aldrich) for 10 h. Then, the agarose beads were washed three times in 0.5 mL of buffer W (50 mM HEPES pH 7.4, 100 mM NaCl, 1.96 mM DDM). The protein was eluted twice or three times with 10 µL per transfected dish with Buffer W supplemented with 0.2 mg/mL FLAG peptide for 8 h at 4°C with gentle mixing. Finally, the obtained target protein was quantified by BCA (Thermo).

### Dithionite assay

For preparation of liposomes and proteo-liposomes using a glass syringe, add 1, 435 µL POPC (25 mg/mL, in chloroform) and 160 µL POPG (25 mg/mL, in chloroform) to a round bottom flask to obtain 52.5 µM lipids in a molar ratio of POPC:POPG = 9:1. Then dry the lipid overnight using a rotary evaporator. Next, the resulting lipid membrane was hydrated in buffer A (10 mL of 50 mM HEPES pH 7.4, 100 mM NaCl), gently mixed for 1–2 h, and ultrasonicated for 10 min at room temperature with a frequency of 40 kHz. The liposomes were then extruded 11 times through a 400 nm pore size membrane and 5 times through a 200 nm pore size membrane. For scramblase activity assay, pipette 800 µL of LUVs into 2 mL microfuge tube, add 5.3 µL of buffer A and 34.7 µL of 10% (w/v) DDM dissolved in Buffer A, and incubate for 3 h at room temperature with end-over-end mixing. After 3 h of vesicle destabilization add the dissolved NBD-labeled phospholipid, the liposomes were incubated with 40 µL (from a concentration of 1 μg/μL as detected by BCA) of purified TgDAT1 protein in 0.1%DDM and 45 µL of NBD-C6-PC (Avanti #810130P) for 1 h, and then 80 mg/mL Bio-Beads were added and incubated at room temperature for 1 h. The samples were again added with 160 mg/mL Bio-Beads and incubated at room temperature for 2 h to remove the detergent. The sample is then transferred to a new tube containing 160 mg/mL of fresh Bio-Beads. The sample is rotated overnight at 4°C. Liposomes (50 µL) containing NBD-labeled lipids were added to 1, 950 µL of buffer A and mixed. Add 40 µL of the 1 M dithionite solution (Macklin, #S817916) to the cuvette 50 s after starting the fluorescence recording and continue to record fluorescence for an additional 600 s using an EnVision plate reader (PerkinElmer) with an excitation wavelength of 470 nm and emission wavelength of 530 nm.

### Plaque assay

For the plaque assays, 500 parasites were inoculated into each well of 12-well plates containing a BJ-5ta monolayer and cultured with Rapa/IAA. After 24 h, the plates were supplemented with 2 mL of fresh medium, and the cells were continuously cultured for an additional 8 days. The cells were then immobilized overnight and stained with Coomassie Brilliant Blue G250 (Beyotime, ST030) for 3 h.

### Intracellular replication assay

TgVPS13A-LoxP parasites (1x10^5^) were treated with Rapa after 72 h,and were inoculated into a 12-well plate containing a BJ-5ta monolayer. After 3 h, the parasites that did not successfully invade were washed off with warm PBS. Following this, the samples were treated with Rapa for 24 h. The parasites were then fixed, stained with rabbit anti-TgSAG2 and Alexa Fluor 488-coupled anti-rabbit IgG at room temperature for 1 h and detected using IFA. Finally, the number of parasites was counted under a fluorescent microscope (Zeiss Axio Vert.A1).Results are from three independent biological replicates.

### Oligonucleotides

All primers used in this study are listed in S2 Table.

### Statistical analysis

Data were analyzed using GraphPad Prism 9 software. The results are presented as the mean ± SD or mean ± SEM from triplicate, parallel, independent experiments, unless stated otherwise. All statistical analyses were conducted using either the student’s t-test or a two-way ANOVA, unless specified differently.

## Supporting information

S1 Fig Identification of VPS13 family proteins in *T. gondii.* **(A)** Alphafold-predicted and manually curated structure of TgVPS13A. **(B)** A schematic diagram of inserting a SmFP-HA tag at the C-terminal of TgVPS13A. **(C)** PCR analysis confirming the SmFP-HA insertion at the C-terminal of TgVPS13A.
(TIF)

S2 Fig Identification of VAP family protein in *T. gondii.* **(A)** Phylogenetic analysis of TgVAP was conducted using alignment with MAFFT, trimming with trimAI, and construction of the phylogenetic tree by IQtree with the maximum likelihood method of 1000 bootstrap replicates. The final analysis results are visualized by iTOL. Boostrap values have been added near the nodes of the tree. If the Bootstrap value > 70, then this branch is reliable. **(B)** Multialignment of TgVAP with its human orthologues by Clustal Omega. **(C)** Surface view of HADDOCK modelled complex between TgVPS13A (N term 900–1500aa) and TgVAP showing the top ranked model of the best scoring cluster with TgVPS13A in orange and TgVAP in blue color. **(D)** IFA showing the colocalization of TgSec61β with the ER-resident protein. **(E)** Quantitative analysis of TgVPS13A degradation from 24h to 96h under Rapa treatment using IFA. Data are mean ± SEM from three independent slides, and at least 100 PVs were counted per slide. **(F)** Western blotting was used to quantify the degradation of TgVAP from 6 h to 24 h under the action of IAA. TgSAG1-GFP-HDEL. Magenta: mouse anti-V5; Green: EGFP signal; Blue: DAPI. Scale bars: 2 μm (merged panels) and 0.2 μm (zoomed panels).
(TIF)

S3 Fig A global screening of annotated transporters in ToxoDB. **(A)** A global screening of annotated transporters in ToxoDB was conducted by inserting a HA tag endogenously using the CRISPR/Cas9 method. **(B)** IFA showing the localization of 64 proteins.
(TIF)

S4 Fig Phenotypic analysis of IMC assembly in the TgDAT1-deleted parasites. **(A)** Western blot analysis of the expression of the protein PCAGGS-TgDAT1/TgDAT1-mut-3Flag used for lipid flipping.The protein used for examination of the scramblase activity was 40 μL (from a concentration of 1 μg/μL as detected by BCA). **(B)** Schematic diagram of inserting a 12 × HA-AID* tag at the N-terminal of TgDAT1. **(C)** Immunofluorescence analysis was conducted to assess TgDAT1 expression in the parasite, both in the presence and absence of Indole-3-acetic acid (IAA). The parasites were cultured with or without IAA for 24 h and stained with antibodies against TgSAG2 and HA. **(D)** Western blotting was used to quantify the degradation of TgDAT1 from 6 h to 24 h with and without IAA. **(E)** The biogenesis of the early IMC was investigated by staining with localization of TgAC9 in TgDAT1-deletion strain. Parasites were transfected with plasmids expressing TgAC9–3 × MYC under the control of the GRA1 promoter. The abnormal and normal PVs were counted. Data are mean ± SEM from three independent slides, and at least 100 PVs were counted per slide. The difference in the number of abnormal PVs was statistically analyzed by unipaired t test; ****P < 0.0001. **(F)** Colocalization of TgAC9 and TgIMC29 in atrophic daughter IMCs in the TgDAT1 deletion strain. A schematic diagram of TgIMC29 colocalization with TgAC9 on atrophic daughter IMCs in TgDAT1-deficient parasites is shown at the right side. Magenta: mouse anti-TgSAG2 polyclonal antibodies; Green: rabbit anti-HA antibody; Blue: DAPI. Scale bars: 2 μm.
(TIF)

S5 Fig Domain analysis of VPS13 candidates in *T. gondii.* **(A)** Domain analysis of the VPS13 family in humans and yeast. **(B)** Schematic diagrams of the domain architecture of VPS13 candidates.
(TIF)

S6 Fig Phylogenetic analysis of other VPS13 candidates in *T. gondii.* **(A)** Phylogenetic analysis of the VPS13 family. The phylogeny of VPS13 sequences was reconstructed using maximum likelihood (ML) analysis. Sequences were aligned with MAFFT and trimmed with trimAI, and the tree was inferred using IQ-TREE with 1,000 bootstrap replicates. The final tree was visualized using iTOL. Bootstrap values are displayed at the nodes, with values > 70 indicating high branch reliability. **(B)** Phylogenetic analysis of the VPS13 family was conducted using MEGA12. Multiple sequence alignment was performed with the ClustalW algorithm, and the phylogenetic tree was reconstructed using the Neighbor-Joining (NJ) method. Branch reliability was assessed with 1,000 bootstrap replicates.
(TIF)

S7 Fig Phylogenetic analysis of the N-terminal domain of VPS13 sequences. Sequences were aligned with MAFFT, trimmed with trimAI, and the phylogenetic tree was constructed using IQ-TREE (ML method) with 1,000 bootstrap replicates. Results were visualized in iTOL. Bootstrap values are shown at the nodes; values > 70 indicate robust branch support.
(TIF)

S8 Fig TgVAP-TgVPS13A-TgDAT1 forms a complex for lipid targeting to the nascent IMC. **(A)** Co-immunoprecipitation assays (Co-IPs) of 3 × HA-tagged TgVPS13A-VAB with 3 × FLAG-tagged full-length or N terminal-deleted (1–303aa) version of TgDAT1. Cells were transiently transfected with plasmids expressing 3 × FLAG-tagged TgVPS13A-VAB and 3 × HA-tagged TgDAT1 or its mutant. **(B)** Surface view of HADDOCK modelled complex between TgVPS13A-VAB and TgDAT1 showing the top ranked model of the best scoring cluster with TgVPS13A-VAB in magenta and TgDAT1 in vermillion color is shown between the western blotting images. **(C)** Co-IP was performed using 3 × FLAG-tagged TgVAP and 3 × HA-tagged TgVPS13A-N928-1392aa. Cells were transiently transfected with plasmids expressing 3 × FLAG-tagged TgVAP and 3 × HA-tagged TgVPS13A-N928-1392aa. The resulting cell lysates were subjected to immunoprecipitation and then blotted with anti-FLAG and anti-HA antibodies. **(D)** IFA showing the colocalization of TgVPS13A&TgVAP, TgVPS13A&TgDAT1 in IMC budding. In TgVPS13A-SMFP-HA parasites, a 4 × MYC tag or an EGFP tag was endogenously inserted into the C-terminus of TgDAT1 or the N-terminus of TgVAP respectively. The MOC was analyzed with mean  ±  SEM by ImageJ. The MOC shown were the averages and standard error of the mean. This MOC includes tM1 and tM2. tM1 is above autothreshold of TgVAP/TgVPS13A. tM2 is above autothreshold of TgVPS13A/TgDAT1. “N” represents the number of parasites. Magenta: rabbit anti-HA, mouse anti-MYC; Green: rabbit anti-HA, EGFP signal; Blue: DAPI. Scale bars: 2 μm (merged panels) and 0.2 μm (zoomed panels). **(E)** Full length of TgVPS13A with TgVAP and TgDAT1 fitted in the interacting pockets identified with HADDOCK. Dark Blue: TgVAP, Dark orange:TgDAT1, Green: Choerin domain (Pfam: PF12624), Magenta: VAB domain (Pfam: PF25036), Blue: ATG2_C domain (Pfam: PF13329). (F) IFA showing the colocalization of TgDAT1, TgVPS13A, and TgVAP at the early and late stages of IMC budding. Magenta: rabbit anti-HA; Green: EGFP signal; Blue: mouse anti-MYC. Scale bars: 2 μm (merged panels) and 0.2 μm (zoomed panels).
(TIF)

S1 Table A list of 71 annotated putative transporters. This table presents the geneID, comments, Phenotype Scores and transmembrane domains of 71 putative transporters.
(DOCX)

S2 Table List of all primers used in this study. (DOCX)

S3 Table The list of all guide RNA sequences. The table presents the specific guide RNA sequences of TgVPS13A (TGGT1_291180), TgVAP (TGGT1_318160), TgDAT1 (TGGT1_258700), TgIMC29 (TGGT1_243200), TgGAPM3 (TGGT1_271970), TgSec61β (TGGT1_211040) and TgSec13 (TGGT1_201700).
(DOCX)

S1 File This text describes the methods used for pairwise co-immunoprecipitation, molecular docking, co-localization, and complex modeling. (DOC)

S2 File This file contains the original, uncropped, and unadjusted images underlying all blot and gel results reported in this study (corresponding to Figs 4D, 6B, S2F, S4A, S4D, S7A, and S7C). (PDF)
