# Supplementary figures and images for: An ER-IMC bridge protein TgVPS13A and an IMC-resident scramblase TgDAT1 drive daughter budding in Toxoplasma gondii
Source: PLoS Pathog. 2026 Jun 18;22(6):e1013865. doi: 10.1371/journal.ppat.1013865 (PMC13298984; doi:10.1371/journal.ppat.1013865)

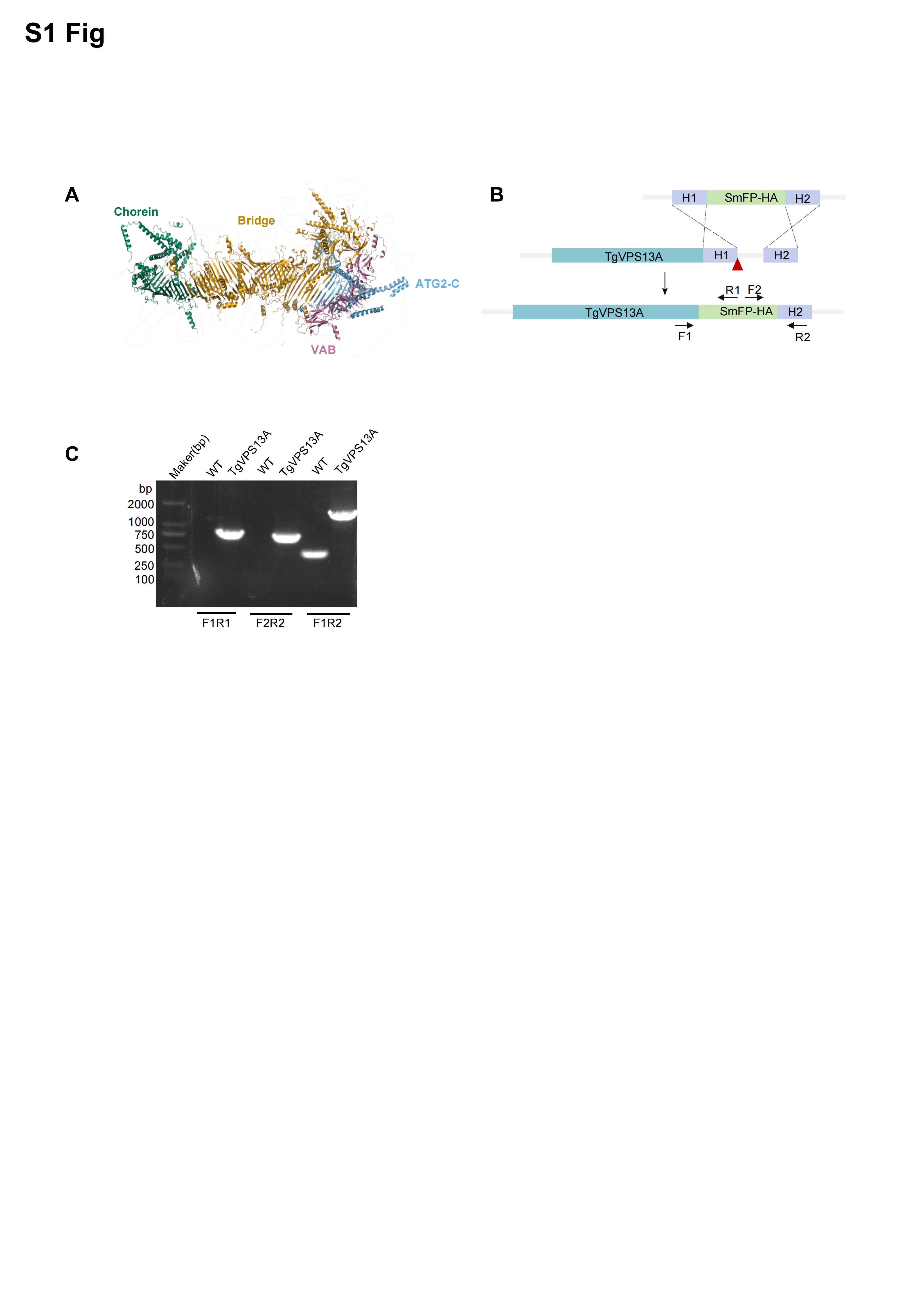

Supplement: S1 Fig — (A) Alphafold-predicted and manually curated structure of TgVPS13A. (B) A schematic diagram of inserting a SmFP-HA tag at the C-terminal of TgVPS13A. (C) PCR analysis confirming the SmFP-HA insertion at the C-terminal of TgVPS13A. (TIF) [file ppat.1013865.s001.tif]

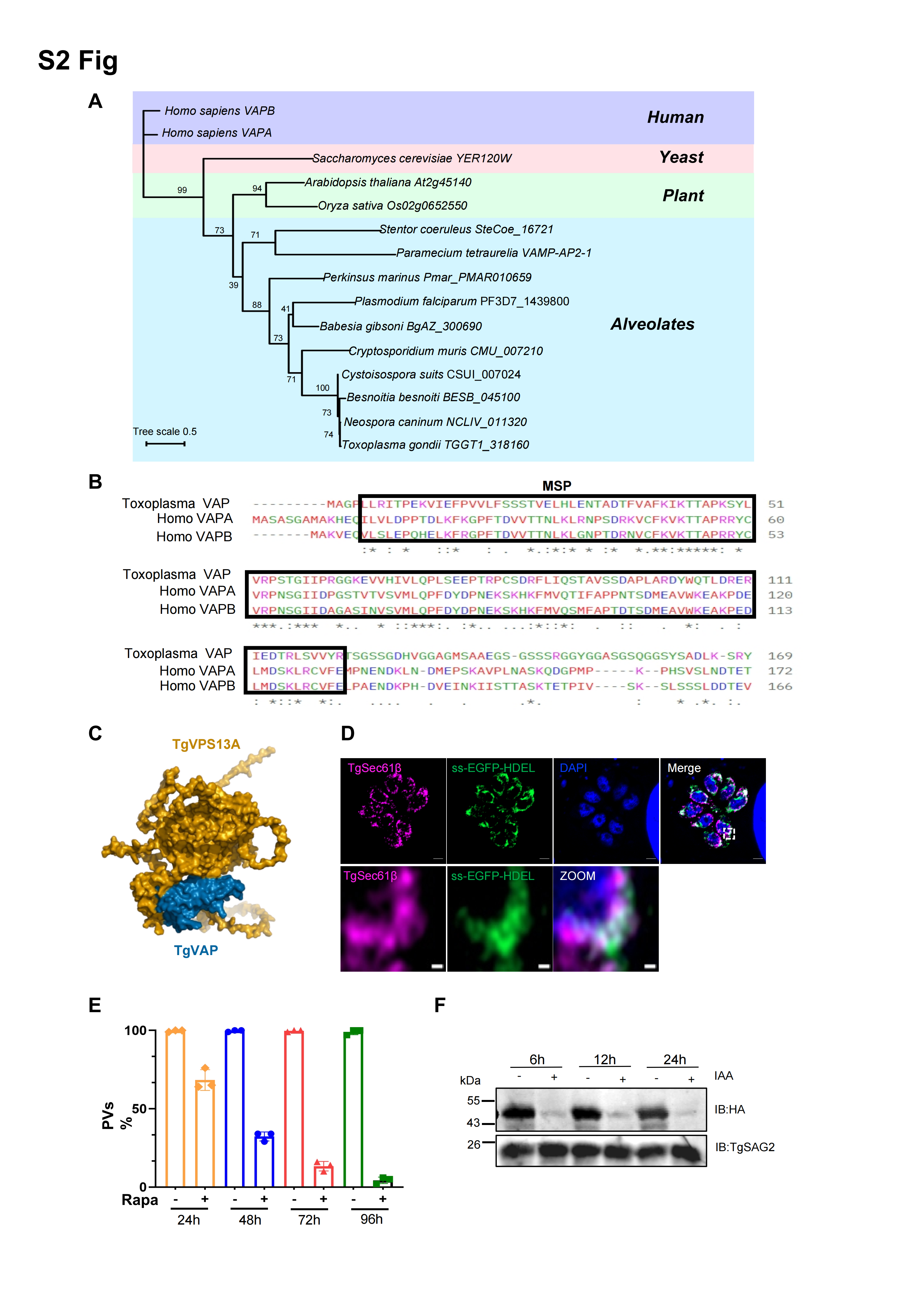

Supplement: S2 Fig — (A) Phylogenetic analysis of TgVAP was conducted using alignment with MAFFT, trimming with trimAI, and construction of the phylogenetic tree by IQtree with the maximum likelihood method of 1000 bootstrap replicates. The final analysis results are visualized by iTOL. Boostrap values have been added near the nodes of the tree. If the Bootstrap value > 70, then this branch is reliable. (B) Multialignment of TgVAP with its human orthologues by Clustal Omega. (C) Surface view of HADDOCK modelled complex between TgVPS13A (N term 900–1500aa) and TgVAP showing the top ranked model of the best scoring cluster with TgVPS13A in orange and TgVAP in blue color. (D) IFA showing the colocalization of TgSec61β with the ER-resident protein. (E) Quantitative analysis of TgVPS13A degradation from 24h to 96h under Rapa treatment using IFA. Data are mean ± SEM from three independent slides, and at least 100 PVs were counted per slide. (F) Western blotting was used to quantify the degradation of TgVAP from 6 h to 24 h under the action of IAA. TgSAG1-GFP-HDEL. Magenta: mouse anti-V5; Green: EGFP signal; Blue: DAPI. Scale bars: 2 μm (merged panels) and 0.2 μm (zoomed panels). (TIF) [file ppat.1013865.s002.tif]

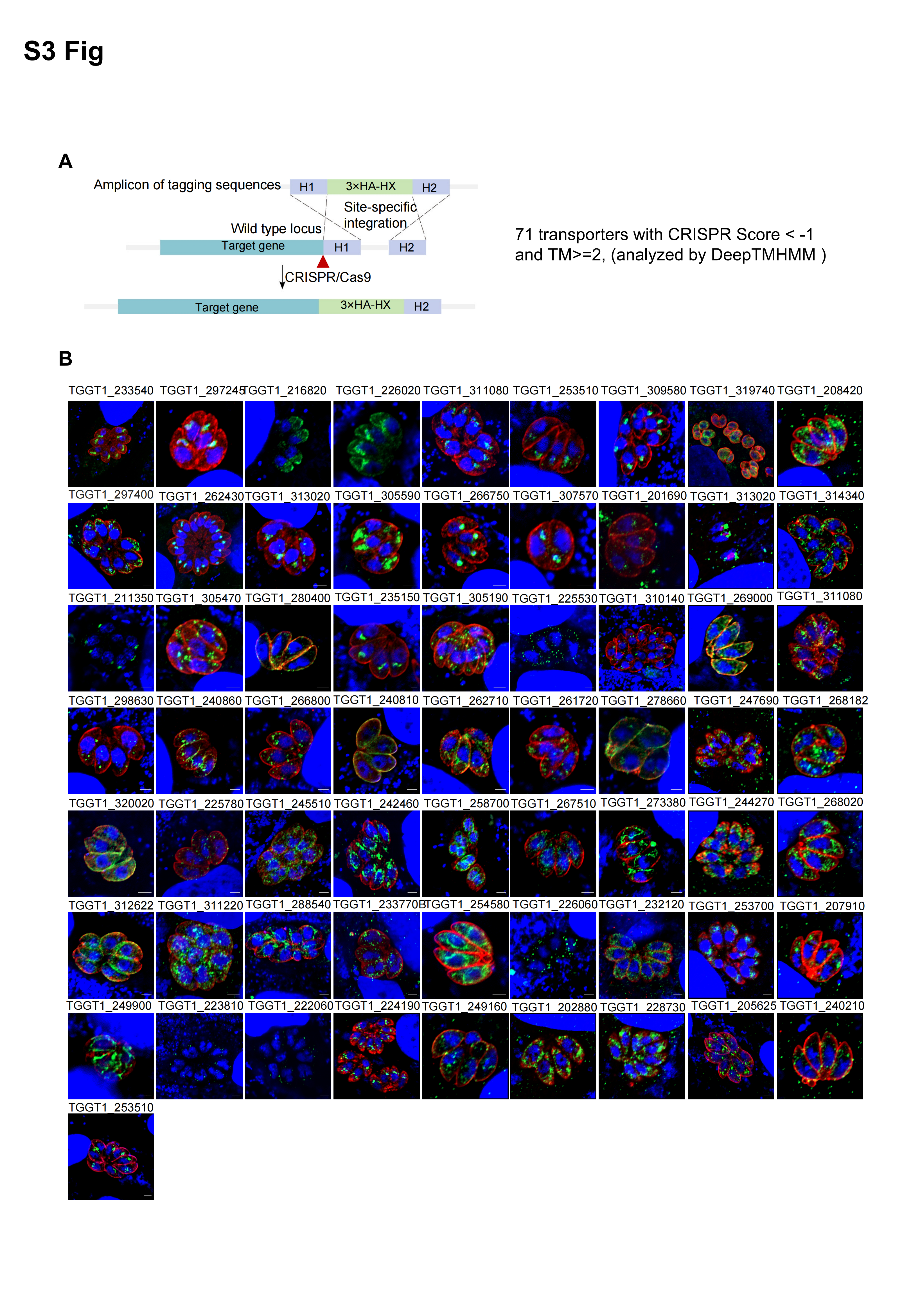

Supplement: S3 Fig — (A) A global screening of annotated transporters in ToxoDB was conducted by inserting a HA tag endogenously using the CRISPR/Cas9 method. (B) IFA showing the localization of 64 proteins. (TIF) [file ppat.1013865.s003.tif]

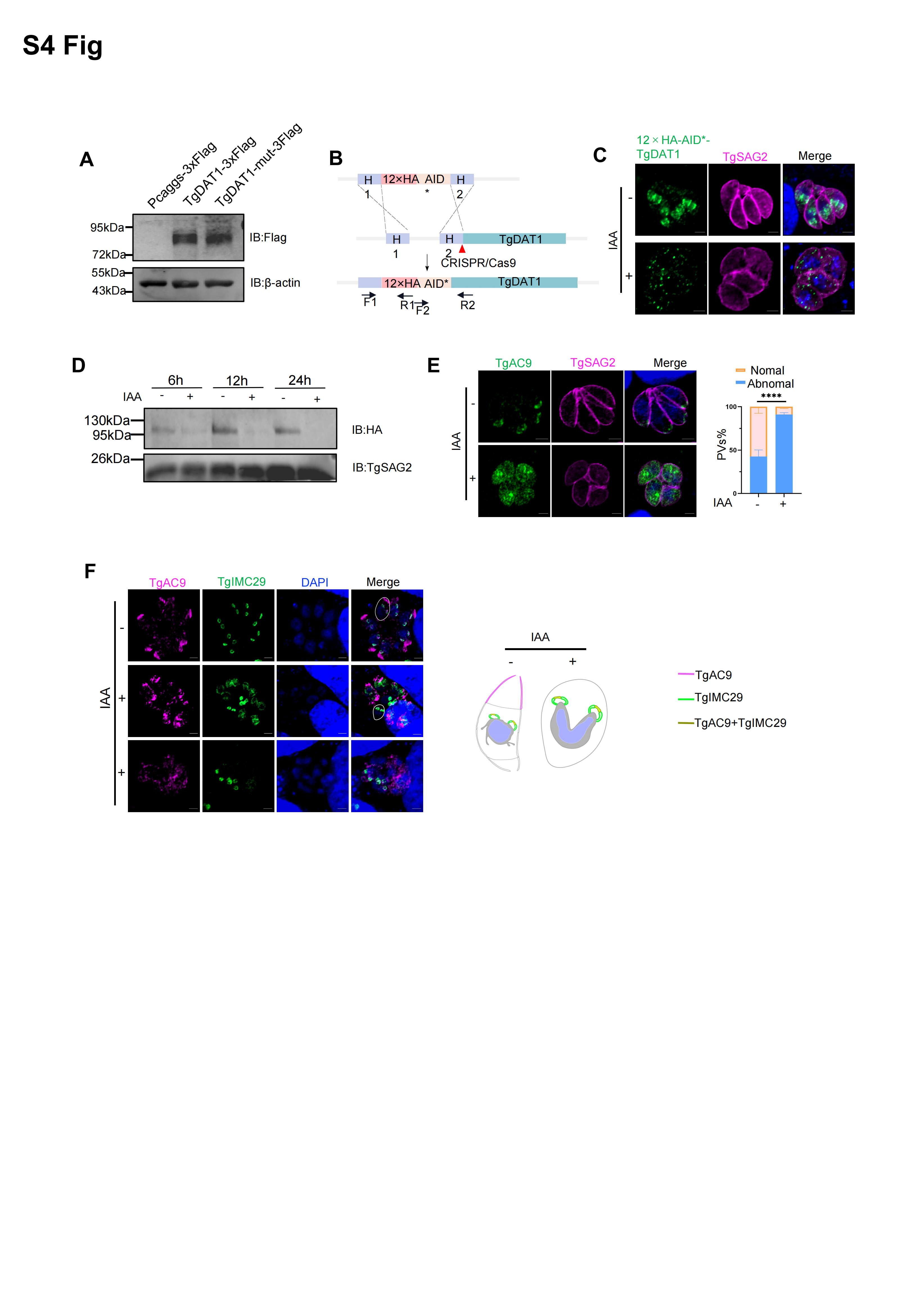

Supplement: S4 Fig — (A) Western blot analysis of the expression of the protein PCAGGS-TgDAT1/TgDAT1-mut-3Flag used for lipid flipping.The protein used for examination of the scramblase activity was 40 μL (from a concentration of 1 μg/μL as detected by BCA). (B) Schematic diagram of inserting a 12 × HA-AID* tag at the N-terminal of TgDAT1. (C) Immunofluorescence analysis was conducted to assess TgDAT1 expression in the parasite, both in the presence and absence of Indole-3-acetic acid (IAA). The parasites were cultured with or without IAA for 24 h and stained with antibodies against TgSAG2 and HA. (D) Western blotting was used to quantify the degradation of TgDAT1 from 6 h to 24 h with and without IAA. (E) The biogenesis of the early IMC was investigated by staining with localization of TgAC9 in TgDAT1-deletion strain. Parasites were transfected with plasmids expressing TgAC9–3 × MYC under the control of the GRA1 promoter. The abnormal and normal PVs were counted. Data are mean ± SEM from three independent slides, and at least 100 PVs were counted per slide. The difference in the number of abnormal PVs was statistically analyzed by unipaired t test; ****P < 0.0001. (F) Colocalization of TgAC9 and TgIMC29 in atrophic daughter IMCs in the TgDAT1 deletion strain. A schematic diagram of TgIMC29 colocalization with TgAC9 on atrophic daughter IMCs in TgDAT1-deficient parasites is shown at the right side. Magenta: mouse anti-TgSAG2 polyclonal antibodies; Green: rabbit anti-HA antibody; Blue: DAPI. Scale bars: 2 μm. (TIF) [file ppat.1013865.s004.tif]

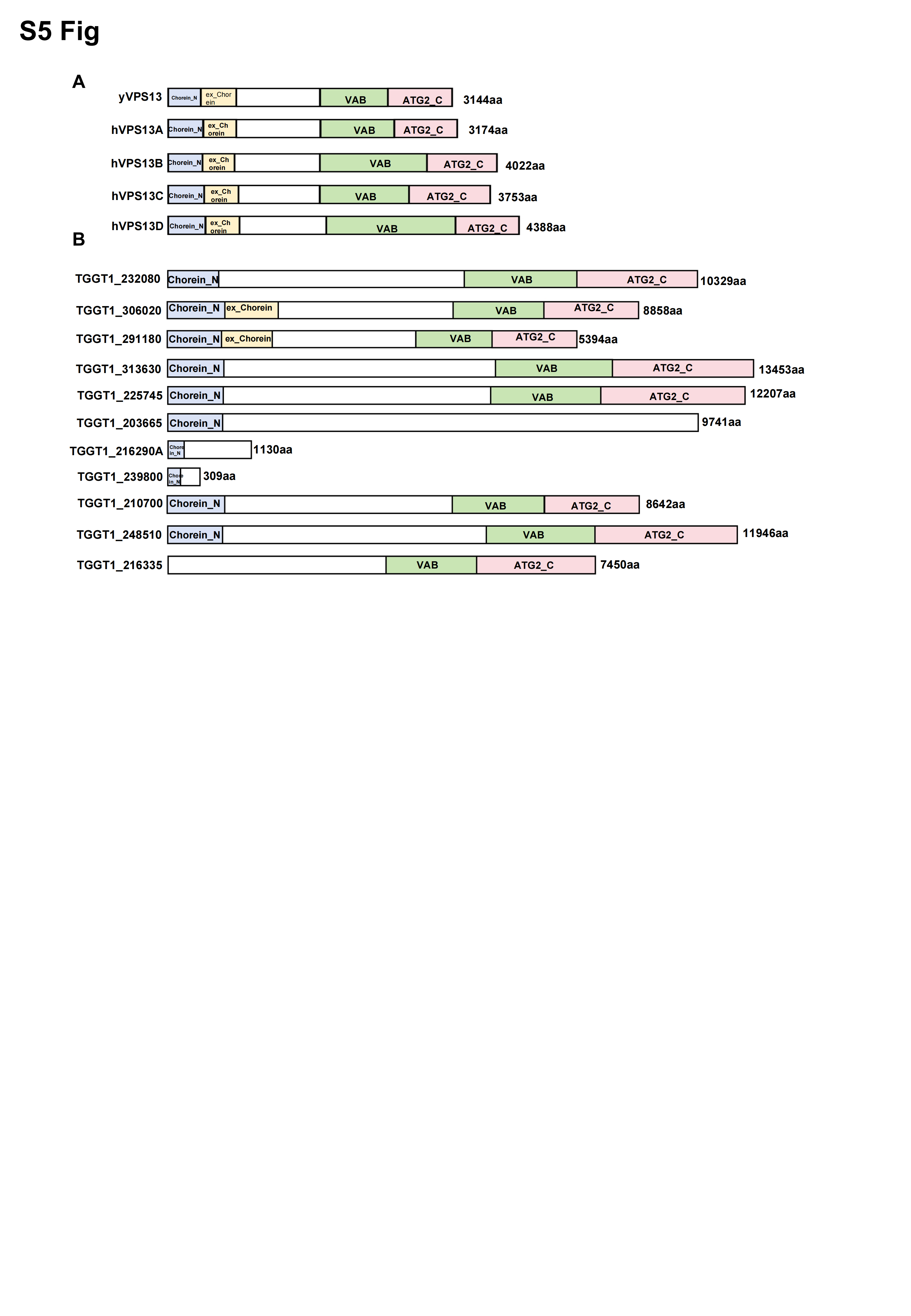

Supplement: S5 Fig — (A) Domain analysis of the VPS13 family in humans and yeast. (B) Schematic diagrams of the domain architecture of VPS13 candidates. (TIF) [file ppat.1013865.s005.tif]

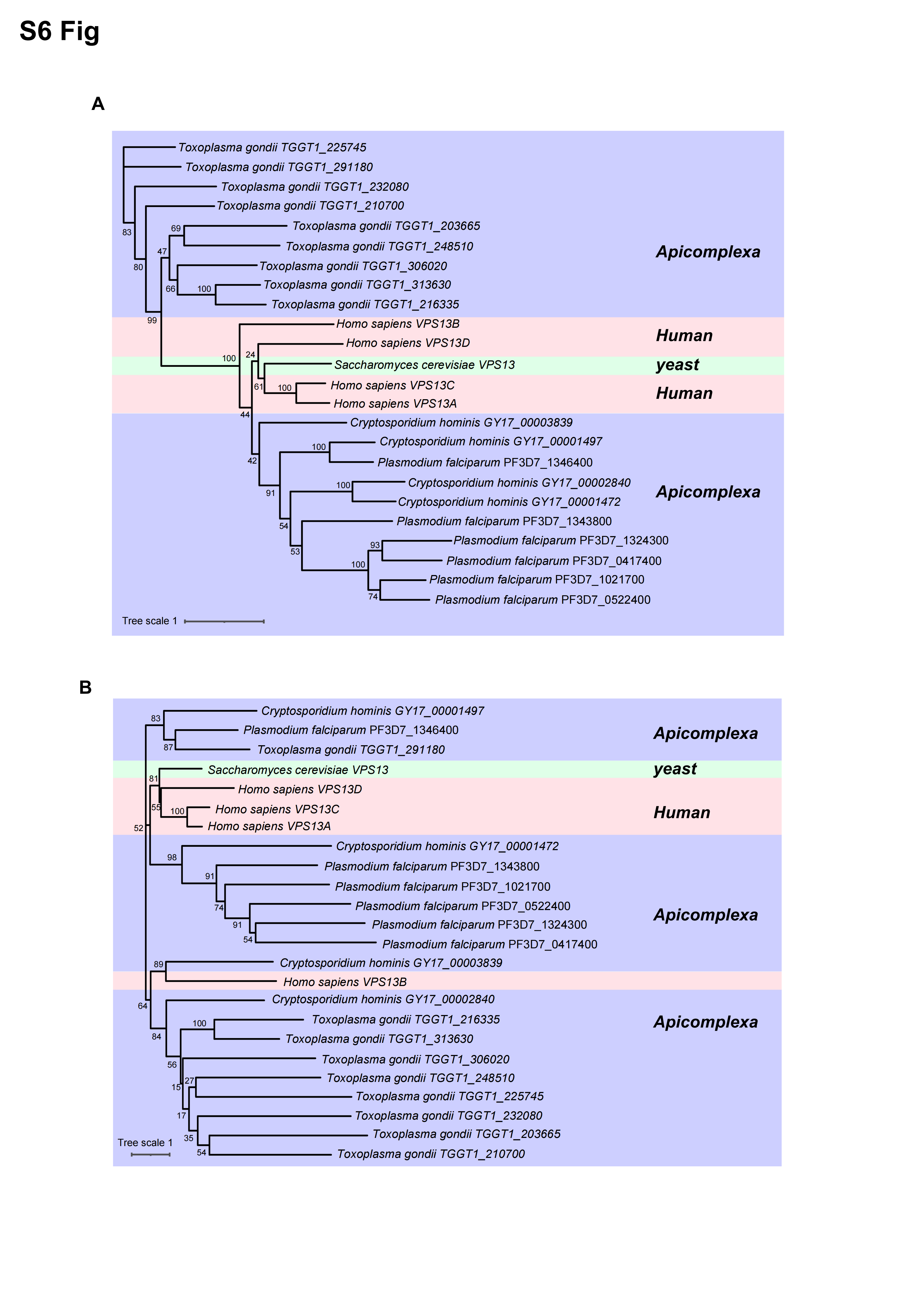

Supplement: S6 Fig — (A) Phylogenetic analysis of the VPS13 family. The phylogeny of VPS13 sequences was reconstructed using maximum likelihood (ML) analysis. Sequences were aligned with MAFFT and trimmed with trimAI, and the tree was inferred using IQ-TREE with 1,000 bootstrap replicates. The final tree was visualized using iTOL. Bootstrap values are displayed at the nodes, with values > 70 indicating high branch reliability. (B) Phylogenetic analysis of the VPS13 family was conducted using MEGA12. Multiple sequence alignment was performed with the ClustalW algorithm, and the phylogenetic tree was reconstructed using the Neighbor-Joining (NJ) method. Branch reliability was assessed with 1,000 bootstrap replicates. (TIF) [file ppat.1013865.s006.tif]

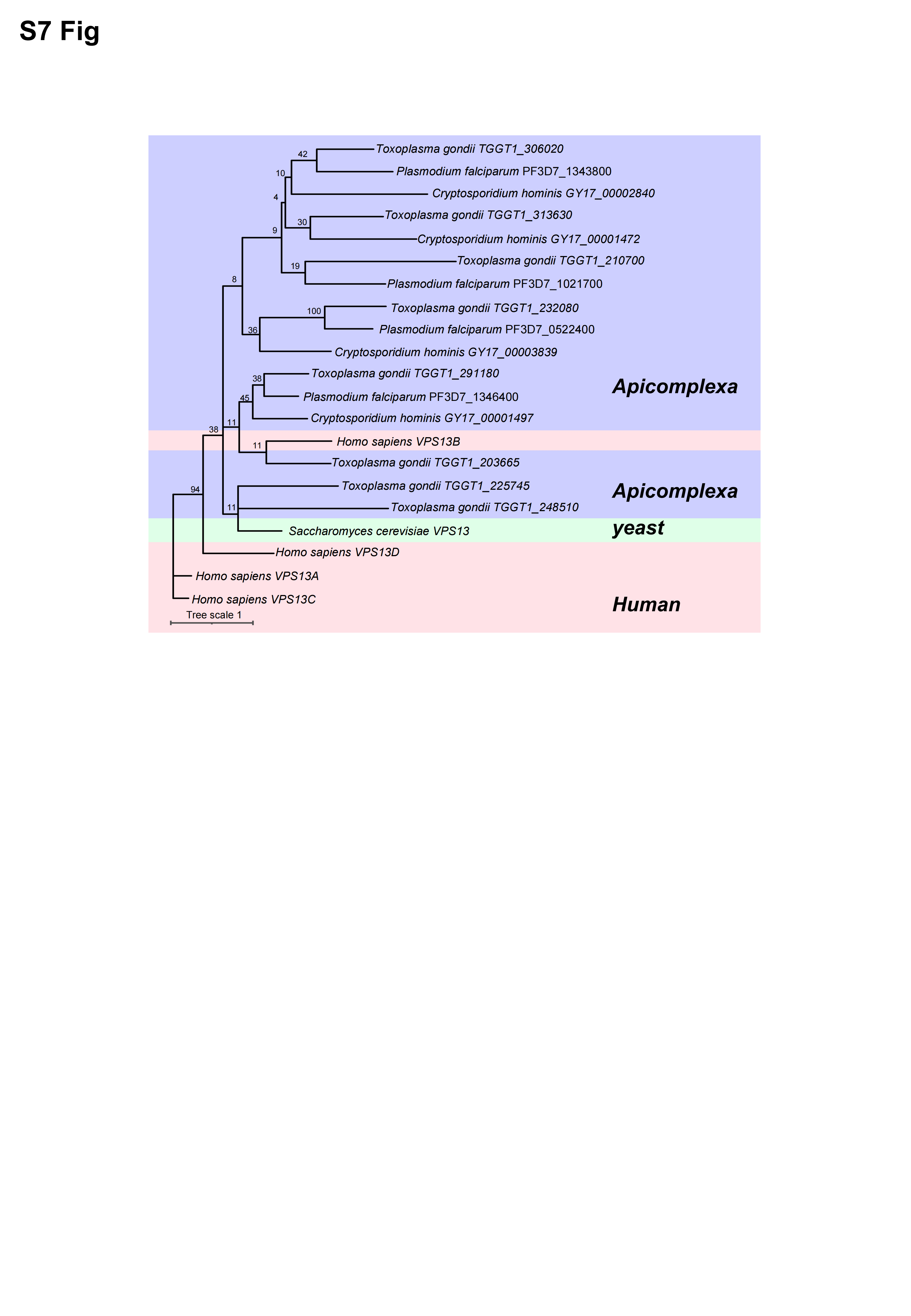

Supplement: S7 Fig — Sequences were aligned with MAFFT, trimmed with trimAI, and the phylogenetic tree was constructed using IQ-TREE (ML method) with 1,000 bootstrap replicates. Results were visualized in iTOL. Bootstrap values are shown at the nodes; values > 70 indicate robust branch support. (TIF) [file ppat.1013865.s007.tif]

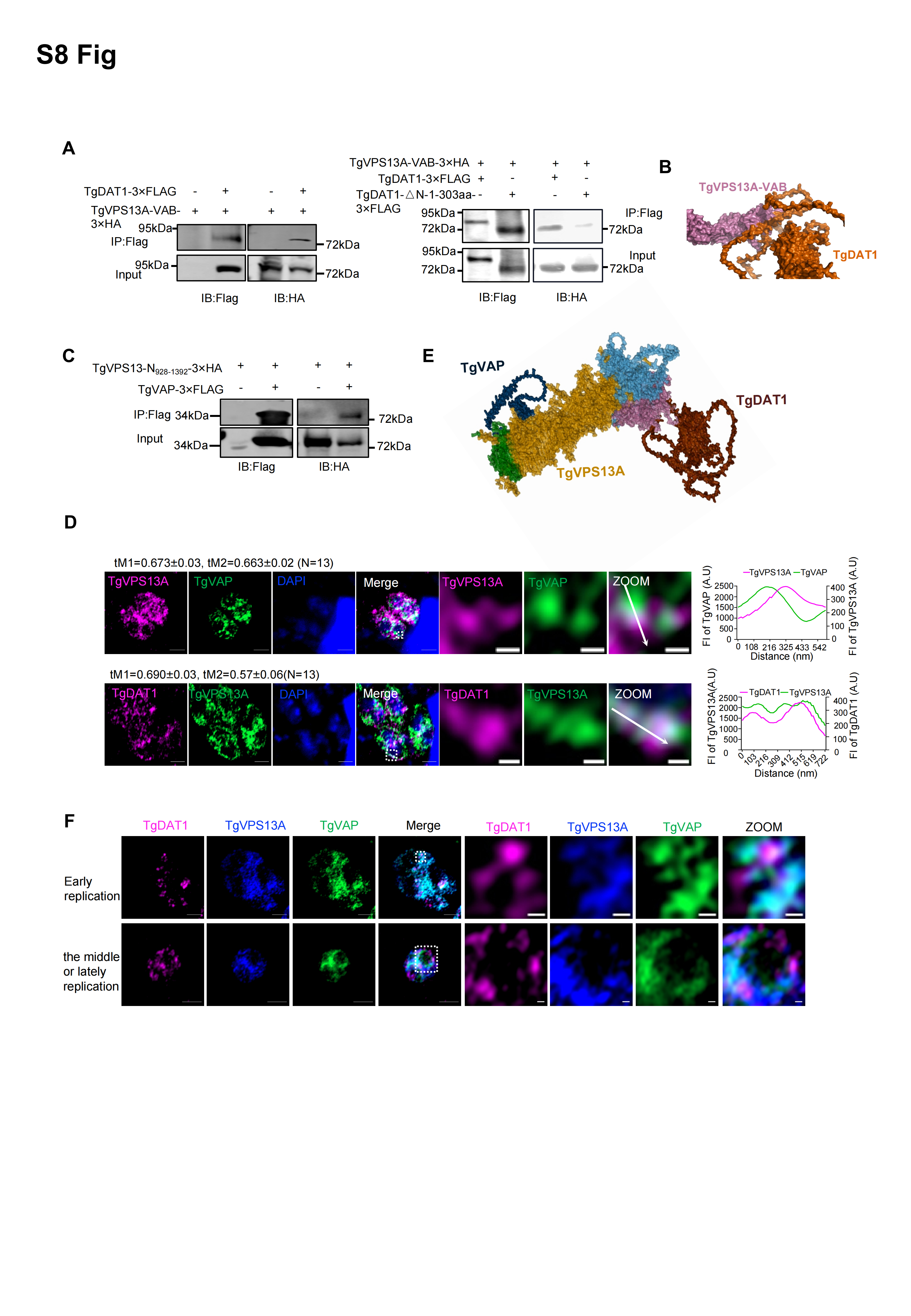

Supplement: S8 Fig — (A) Co-immunoprecipitation assays (Co-IPs) of 3 × HA-tagged TgVPS13A-VAB with 3 × FLAG-tagged full-length or N terminal-deleted (1–303aa) version of TgDAT1. Cells were transiently transfected with plasmids expressing 3 × FLAG-tagged TgVPS13A-VAB and 3 × HA-tagged TgDAT1 or its mutant. (B) Surface view of HADDOCK modelled complex between TgVPS13A-VAB and TgDAT1 showing the top ranked model of the best scoring cluster with TgVPS13A-VAB in magenta and TgDAT1 in vermillion color is shown between the western blotting images. (C) Co-IP was performed using 3 × FLAG-tagged TgVAP and 3 × HA-tagged TgVPS13A-N928-1392aa. Cells were transiently transfected with plasmids expressing 3 × FLAG-tagged TgVAP and 3 × HA-tagged TgVPS13A-N928-1392aa. The resulting cell lysates were subjected to immunoprecipitation and then blotted with anti-FLAG and anti-HA antibodies. (D) IFA showing the colocalization of TgVPS13A&TgVAP, TgVPS13A&TgDAT1 in IMC budding. In TgVPS13A-SMFP-HA parasites, a 4 × MYC tag or an EGFP tag was endogenously inserted into the C-terminus of TgDAT1 or the N-terminus of TgVAP respectively. The MOC was analyzed with mean ± SEM by ImageJ. The MOC shown were the averages and standard error of the mean. This MOC includes tM1 and tM2. tM1 is above autothreshold of TgVAP/TgVPS13A. tM2 is above autothreshold of TgVPS13A/TgDAT1. “N” represents the number of parasites. Magenta: rabbit anti-HA, mouse anti-MYC; Green: rabbit anti-HA, EGFP signal; Blue: DAPI. Scale bars: 2 μm (merged panels) and 0.2 μm (zoomed panels). (E) Full length of TgVPS13A with TgVAP and TgDAT1 fitted in the interacting pockets identified with HADDOCK. Dark Blue: TgVAP, Dark orange:TgDAT1, Green: Choerin domain (Pfam: PF12624), Magenta: VAB domain (Pfam: PF25036), Blue: ATG2_C domain (Pfam: PF13329). (F) IFA showing the colocalization of TgDAT1, TgVPS13A, and TgVAP at the early and late stages of IMC budding. Magenta: rabbit anti-HA; Green: EGFP signal; Blue: mouse anti-MYC. Scale bars: 2 μm (merged panels) [file ppat.1013865.s008.tif]

Fig 4D

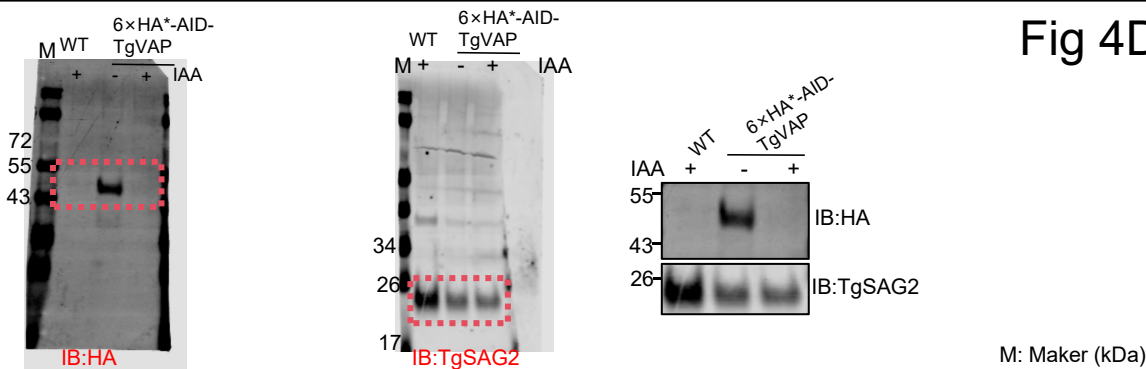

Fig 6B

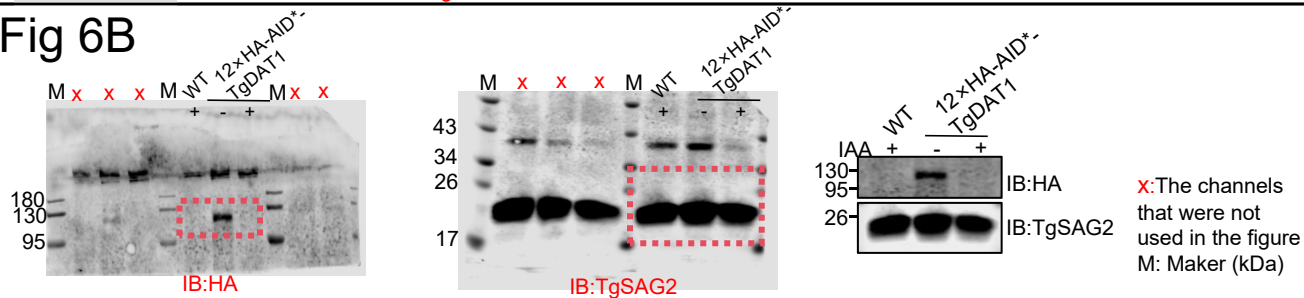

Fig S2F

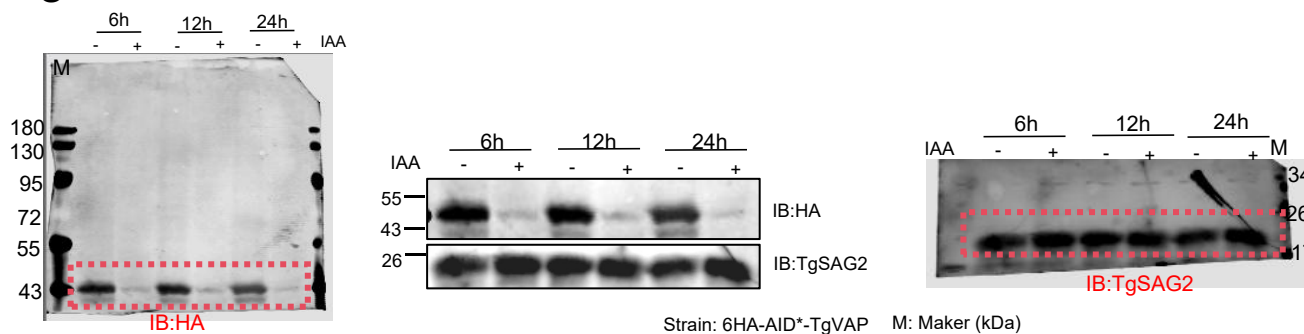

Fig S4A

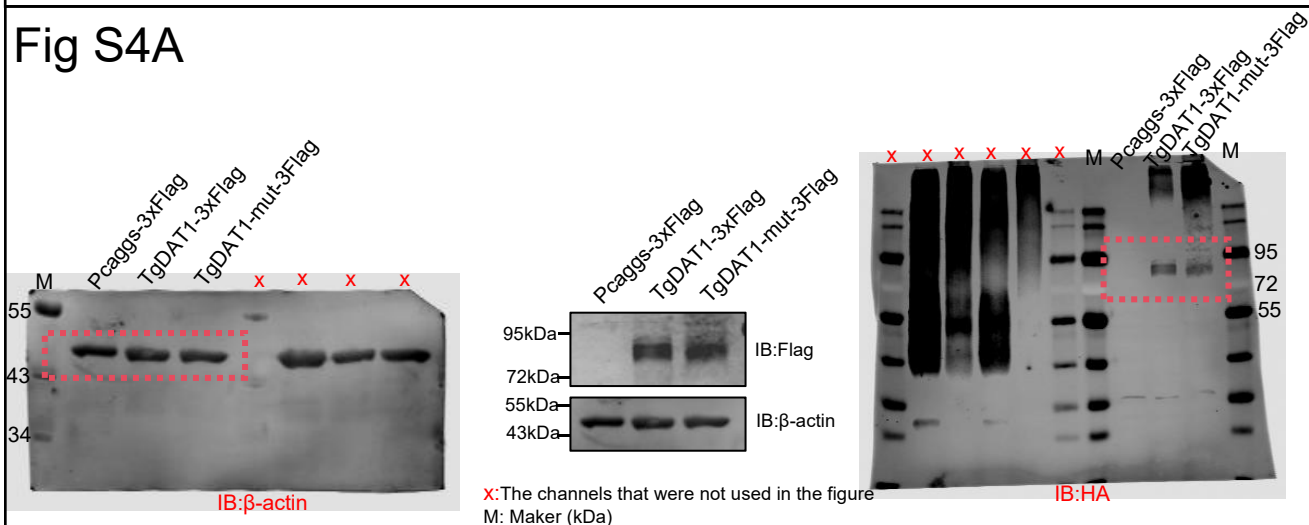

Fig S4D

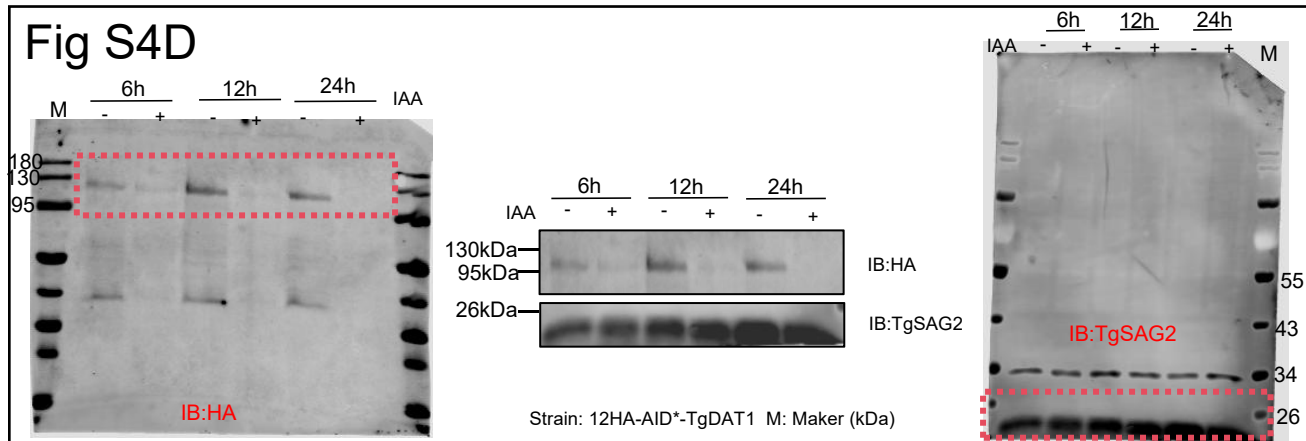

Fig S7A

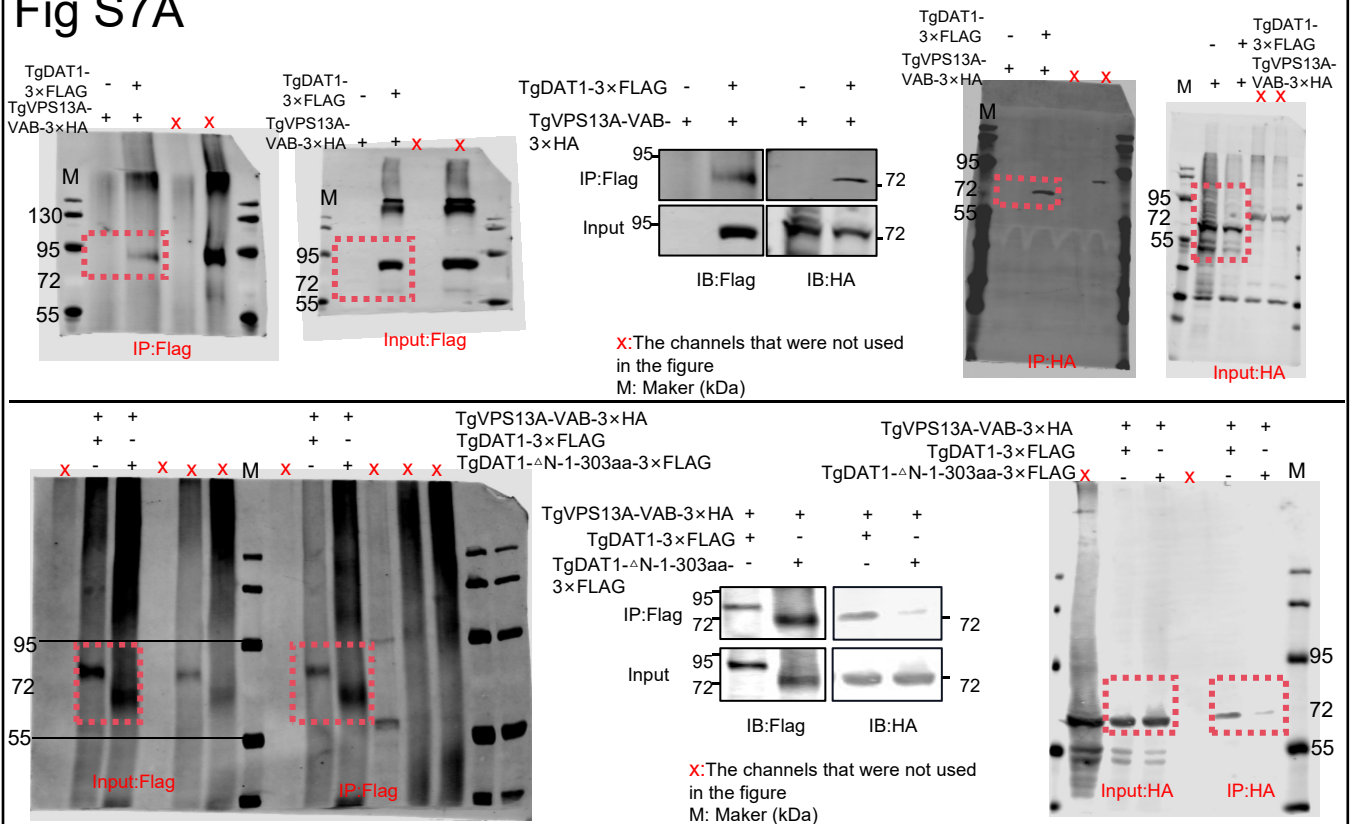

Fig S7C

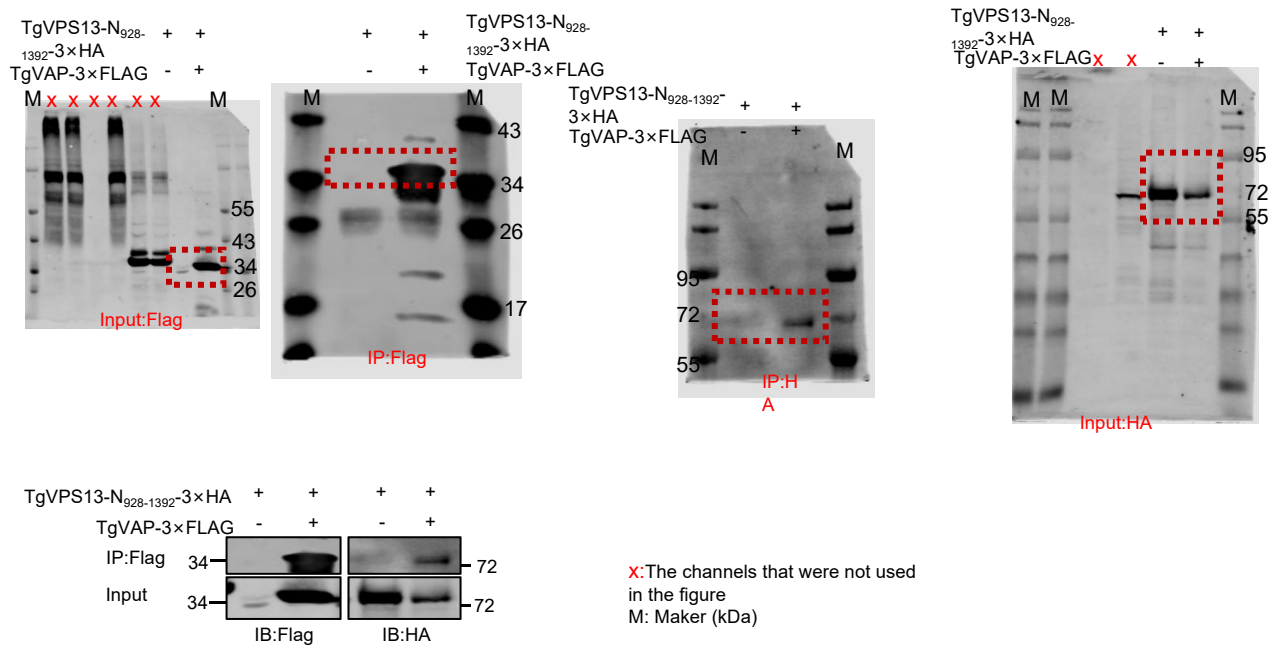

Supplement: S2 File — (PDF) [file ppat.1013865.s013.pdf]
