# Supplementary material for: An ER-IMC bridge protein TgVPS13A and an IMC-resident scramblase TgDAT1 drive daughter budding in Toxoplasma gondii
Source: PLoS Pathog. 2026 Jun 18;22(6):e1013865. doi: 10.1371/journal.ppat.1013865 (PMC13298984; doi:10.1371/journal.ppat.1013865)
Supplement: S1 Table — This table presents the geneID, comments, Phenotype Scores and transmembrane domains of 71 putative transporters. (DOCX) [file ppat.1013865.s009.docx]

**S1 Table**

| Gene ID | Comment | Phenotype Score | TM domain |
| --- | --- | --- | --- |
| TGGT1_201690 | BT1 family protein Folate/biopterin transporter | -1.15 | 14 |
| TGGT1_202880 | mitochondrial carrier superfamily protein | -3.99 | 2 |
| TGGT1_205625 | transporter, major facilitator family protein | -2.05 | 12 |
| TGGT1_207910 | putative manganese resistance 1 protein（calcium/proton exchanger CAX） | -2.77 | 11 |
| TGGT1_208050 | putative ABC transporter | -2.31 | 12 |
| TGGT1_208420 | Sodium:neurotransmitter symporter family protein | -2.26 | 14 |
| TGGT1_211350 | CBS domain-containing protein Metal transporter | -2.18 | 3 |
| TGGT1_216820 | transporter, major facilitator family protein （MFS_spinster_like) | -4.04 | 12 |
| TGGT1_220340 | organic solute transporter ostalpha protein | -1.25 | 7 |
| TGGT1_222060 | cation channel family transporter | -1.34 | 6 |
| TGGT1_223810 | magnesium transporter NIPA | -1.49 | 10 |
| TGGT1_224190 | cation-transporting atpase family protein | -5.24 | 10 |
| TGGT1_225530 | metal cation transporter, ZIP family protein | -2.94 | 8 |
| TGGT1_226020 | transporter, major facilitator family protein(sugar transport domain-containing protein) | -3.53 | 12 |
| TGGT1_226060 | transmembrane amino acid transporter protein | -1.15 | 11 |
| TGGT1_228680 | mitochondrial carrier superfamily protein | -1.3 | 10 |
| TGGT1_228730 | chloroquine resistance transporter CRT | -2.58 | 12 |
| TGGT1_230420 | sarco/endoplasmic reticulum Ca2+-ATPase | -5.43 | 10 |
| TGGT1_232120 | tranport-associated late exocytosis protein The Calcium-dependent Chloride Channel (Ca-ClC) Family | -2.36 | 12 |
| TGGT1_233540 | transporter, major facilitator family protein(ABC1 family beta-lactamase) | -4.02 | 12 |
| TGGT1_233710 | inorganic anion transporter, sulfate permease (SulP) family protein | -2.18 | 12 |
| TGGT1_233770B | calcium-translocating P-type ATPase, PMCA-type protein | -2.51 | 6 |
| TGGT1_235150 | transporter, major facilitator family protein | -5.1 | 12 |
| TGGT1_235402 | CorA family Mg2+ transporter protein | -1.57 | 2 |
| TGGT1_240210 | putative phosphate transporter family protein | -1.32 | 12 |
| TGGT1_240810 | MFS transporter Fmp42, related | -5.4 | 12 |
| TGGT1_240860 | acyltransferase domain-containing protein MMPL family transporter | -2.54 | 2 |
| TGGT1_242460 | mitochondrial carrier superfamily protein | -3.71 | 4 |
| TGGT1_244270 | ATP-binding cassette G family transporter ABCG87 | -2.12 | 6 |
| TGGT1_245510 | phospholipid-translocating P-type ATPase, flippase subfamily protein | -3.76 | 10 |
| TGGT1_247370 | polycystin cation channel protein | -1.42 | 11 |
| TGGT1_247540 | ATP-binding cassette G family transporter ABCG107 | -2.35 | 6 |
| TGGT1_247690 | phospholipid-translocating P-type ATPase, flippase subfamily protein | -1.39 | 10 |
| TGGT1_249160 | UAA transporter family protein | -4.67 | 10 |
| TGGT1_249900 | putative adenine nucleotide translocator | -3.78 | 5 |
| TGGT1_253510 | transporter/permease protein | -2.01 | 10 |
| TGGT1_253700 | transporter, major facilitator family protein | -1.76 | 12 |
| TGGT1_254580 | UDP-galactose transporter family protein | -4.9 | 10 |
| TGGT1_258700 | transporter, major facilitator family protein | -4.55 | 12 |
| TGGT1_261720 | metal cation transporter, ZIP family protein | -2.62 | 4 |
| TGGT1_262710 | Ctr copper transporter family protein | -2.62 | 4 |
| TGGT1_265500 | chloride transporter, chloride channel (ClC) family protein | -2.68 | 10 |
| TGGT1_266750 | putative transporter/permease protein | -1.01 | 10 |
| TGGT1_266800 | putative integral membrane protein Fe(2+)/Mn(2+) transporter | -1.1 | 5 |
| TGGT1_267510 | endoplasmic reticulum-based factor for assembly of V-ATPase Chloride channel protein | -5.38 | 2 |
| TGGT1_267720 | transporter, cation channel family protein | -3.61 | 6 |
| TGGT1_268020 | transporter, major facilitator family protein | -1.33 | 12 |
| TGGT1_268182 | transporter, major facilitator family protein | -2.68 | 8 |
| TGGT1_269000 | ABC transporter family protein | -4.89 | 6 |
| TGGT1_273380 | Ion transport protein/Ion channel/Calcium-activated BK potassium channel alpha subunit, putative | -2.84 | 8 |
| TGGT1_278660 | putative P-type ATPase4 Calcium-transporting ATPase | -4.77 | 10 |
| TGGT1_278990 | putative phosphate carrier | -2.72 | 4 |
| TGGT1_280400 | D-xylose-proton symporter | -2.37 | 15 |
| TGGT1_288540 | Equilibrative nucleoside transporter domain-containing protein | -3.71 | 11 |
| TGGT1_293200 | membrane magnesium transporter | -2.68 | 3 |
| TGGT1_297245 | transporter, major facilitator family protein (apicoplast monocarboxylate transporter AMT2) | -5.61 | 12 |
| TGGT1_297400 | Cyclic nucleotide gated ion channel | -1.91 | 2 |
| TGGT1_298630 | vacuolar transporter chaperone VTC2 | -3.3 | 3 |
| TGGT1_305190 | CorA family Mg2+ transporter protein | -3.16 | 2 |
| TGGT1_305470 | transmembrane amino acid transporter | -2.84 | 11 |
| TGGT1_305590 | ABC transporter transmembrane region domain-containing protein | -3.05 | 12 |
| TGGT1_309580 | transporter, major facilitator family protein | -3.95 | 12 |
| TGGT1_310140 | putative lysosomal cobalamin transporter | -3.14 | 9 |
| TGGT1_310560 | polycystin cation channel protein | -2.38 | 12 |
| TGGT1_311080 | transporter, cation channel family protein | -4.32 | 14 |
| TGGT1_311220 | sulfite exporter TauE/SafE protein | -1.04 | 10 |
| TGGT1_312622 | DUF803 domain-containing protein NIPA | -4.13 | 10 |
| TGGT1_313020 | SLC26A/SulP transporter domain, STAS domain-containing protein | -3.54 | 12 |
| TGGT1_314340 | Sodium:neurotransmitter symporter family protein | -1.1 | 12 |
| TGGT1_319740 | transporter, major facilitator family protein | -1.01 | 10 |
| TGGT1_320020 | transporter, major facilitator family protein | -4.11 | 12 |

**S1 Table.** A list of 71 annotated putative transporters that the CRISPR score greater than -1.0. This table lists the geneID, comments, Phenotype Scores and transmembrane domains of 71 putative transporters.Using the ToxoDB database, filter for transmembrane proteins with CRISPR score greater than -1.0 and transmembrane domain≥2.
