# Supplementary material for: An ER-IMC bridge protein TgVPS13A and an IMC-resident scramblase TgDAT1 drive daughter budding in Toxoplasma gondii
Source: PLoS Pathog. 2026 Jun 18;22(6):e1013865. doi: 10.1371/journal.ppat.1013865 (PMC13298984; doi:10.1371/journal.ppat.1013865)
Supplement: S2 Table — (DOCX) [file ppat.1013865.s010.docx]

**S2 Table**

| Primers | Sequences（5‘-3’） | Purposes |
| --- | --- | --- |
| CAS9-F | actagttctagagcggGA | pSAG1-U6-TgVPS13A-C-Cas9 |
| TgVPS13A-C-gRNA-R | gctatttctagctctaaaacTGGTCGAGTTAGGCAGTTGGcaacttgacatccccatt |  |
| TgVPS13A-C-SMFP-HA-F | GGAGGCGCAGAGCAGCACCAGTGCaGTGGTCGAaTTAGGCAGcTGGCGAACGCTGCAGTATGCCA | Fragment used for insertion of SmFP-HA tag into TgVPS13A C terminal |
| TgVPS13A-C-SMFP-HA-F1 | cTGGCGAACGCTGCAGTATGCCAAATACCAAGACGAAGATTACCCTTATGATGTGCCC |  |
| TgVPS13A-C-SMFP-HA-R | CCGCCCGACACTGTCAACTTGCTGCGTCTCGTTTCCCTTAAGCTGGGTACCAGCGTAG |  |
| TgVPS13A-C-Iden-F1 | AGTCCCATGTCAAACTGCGG | PCR identification of SmFP-HA insertion in TgVPS13A C terminal |
| TgVPS13A-C-Iden-R2 | TGTGCCACGACTCTCCTGAA |  |
| TgVPS13A-C-Iden-F2 | GTTGAGGGTGGATATCCCTA |  |
| TgVPS13A-C-Iden-R1 | TAGGGATATCCACCCTCAAC |  |
| LoxP-TgVPS13A-5'UTR-F | gcataggtaGCCACCATGGGTACCATAACTTCGTATAGCATACATTATACGAAGTTATGATATCTCAGGAGAgGC | pCAGGS-LoxP-TgVPS13A 5'UTR-LoxP-3×HA |
| LoxP-TgVPS13A-5'UTR-F0 | CGAAGTTATGATATCTCAGGAGAgGCTGCGcACTCTcGGCATCCGCTAGAAAGTT |  |
| TgVPS13A-5'UTR-LoxP-R | TTATtttagatctaaaagggaaGATATCCGTcGAAGAGACAAAGACAA |  |
| LoxP-F | ttcccttttagatctaaaATAA |  |
| 3×HA-R | AGGGAAAAAGATCTgctagcAGCGTAATCTGGAACGTC |  |
| TgVPS13A-LoxP-5'UTR-F | CATACATTATACGAAGTTATGATATCTCTGGCGATTTGCCGTGA | pCAGGS-LoxP-TgVPS13A-5'UTR-intron(third)-LoxP-3×HA |
| TgVPS13A-Intron(third)-LoxP-R | ATtttagatctaaaagggaaGCCGTCTCTGTGCGTGTC |  |
| TgVPS13A-N-5'UTR-gRNA-F | taaatggggatgtcaagttAATCGCCAGAGTCTCAGCGAgttttagagctagaaatag | pSAG1-U6-TgVPS13A-N-Double-Cas9(1st&2nd) |
| TgVPS13A-N-Intron(third)-gRNA-R | gctatttctagctctaaaacCCAGCGGTCGTGAGCGTGATaacttgacatccccatt |  |
| TgVPS13A-N-5'UTR-F | GGGGGCGCCGTGTGCGGCGTCTTTTCGCCTTCGCTGAGACtaGCCACCATGGGTACCA | Fragment used for insertion of LoxP-TgVPS13A-5'UTR-N-Intron(third)-LoxP into TgVPS13A N terminal |
| TgVPS13A-N-Intron(third)-R | AGGAACTCGGTTGTGTGTATCACTCTCACGACTGCTGGCGATGGGTACATATAACTTC |  |
| TgVPS13A-N-Iden-F1' | GAGAACGGGAGGTGTACGTC | PCR identification of LoxP-TgVPS13A-5'UTR-N-Intron(third)-LoxP insertion in TgVPS13A N terminal |
| TgVPS13A-N-Iden-F2' | CCGGGTGTATGTACACTCCA |  |
| TgVPS13A-N-Iden-R1' | TGGAGTGTACATACACCCGG |  |
| TgVPS13A-N-Iden-F3' | CGGCCCGACCTGCTACATGT |  |
| TgVPS13A-N-Iden-R2' | ACATGTAGCAGGTCGGGCCG |  |
| TgVPS13A-N-Iden-F4' | TACGCCTCGTCTGTCACAAA |  |
| TgVPS13A-N-Iden-R3' | TTTGTGACAGACGAGGCGTA |  |
| TgVPS13A-N-Iden-R4' | TAGGGCGAGATGGAAGGAAC |  |
| TgVAP-N-gRNA-R | tatttctagctctaaaacCCATTTTCGCGTTTCAATCAcaacttgacatccccattta | pSAG1-U6-TgVAP-N-Cas9 |
| TgVAP-N-12×HA-AID*-F | TGACGCTCTGGTGCAGTGCGCATGATTGAAACGCGAAGATGTACCCgTACGAcGTCC | Fragment used for insertion of 12×HA-AID* tag into TgVAP N terminal |
| TgVAP-N-12×HA-AID*-R | TCACCTTCTCGGGAGTTATCCGCAGCAACGGTCCCGCagagccacctcctccaccgc |  |
| TgVAP-N-Iden-F1 | CCGTGAGCGTCCTTGCTTCC | PCR identification of 12×HA-AID* insertion in TgVAP N terminal |
| TgVAP-N-Iden-R1 | ggagacccacccccaccc |  |
| TgVAP-N-Iden-F2 | AGATTACGCTagcggtgg |  |
| TgVAP-N-Iden-R2 | TTCCGAGAGCGGCTGCAGGA |  |
| TgDAT1-N-gRNA-R | gctatttctagctctaaaacATGAAATGGAGACCTTCGCCaacttgacatccccattt | pSAG1-U6-TgDAT1-N-Cas9 |
| TgDAT1-N-12×HA-AID*-F | TCTCTTTTGAACATCCGTTTTTCGCTCTGCCAATGAAATGTACCCgTACGAcGTCCCcG | Fragment used for insertion of 12HA-AID* tag into TgDAT1 N terminal |
| TgDAT1-N-12×HA-AID*-R | AGGGCGAGGCGCCAGACTCTCGGGGGCGAAGGTCTCagagccacctcctccaccgc |  |
| TgDAT1-N-Iden-F1 | TCTCTGAGCTAGCCGGGCGA | PCR identification of 12HA-AID* insertion in TgDAT1 N terminal |
| TgDAT1-N-Iden-R1 | ggagacccacccccaccc |  |
| TgDAT1-N-Iden-R2 | CGTACTCTCGACTGAAGGGA |  |
| TgDAT1-N-Iden-F2 | AGATTACGCTagcggtgg |  |
| TgIMC29-C-gRNA-R | tatttctagctctaaaacGACACGGCCTCAATTAAAGGCaacttgacatccccattt | pSAG1-U6-TgIMC29-C-Cas9 |
| TgIMC29-C-EGFP-F | GCTCACGCTCAGGCAGCAGTACCCCGGACACGGCCTCAATgtgagcaagggcgaggag | Fragment used for insertion of EGFP tag into TgIMC29 C terminal |
| TgIMC29-C-EGFP-R | GGCTTTTGACGTTTCCCTCCGACTGGTTTCTCTTCCTTTAGATATCcttgtacagctc |  |
| TgIMC29-C-Iden-F | GGCAGGGATGCCTTCGATTG | PCR identification of EGFP insertion in TgIMC29 C terminal |
| TgIMC29-C-Iden-R | CCTTTGCCTACGCGATGCTG |  |
| GRA-TgISP1-F | AGCTTGATGGGATGGATATCATGGGAGCTGTCAGCTCG | GRA-TgISP1-3MYC |
| GRA-TgISP1-3Myc-R | GAGATGAGTTTCTGCTCGATATCTGCCTTCAGCTTCTTCAA |  |
| GRA-TgIMC29-F | GAAGCTTGATGGGATGGATATCATGGAAGCGTTGCCTGTC | GRA-TgIMC29-3×MYC |
| GRA-TgIMC29-R | AGATGAGTTTCTGCTCGATATCATTGAGGCCGTGTCCGGG |  |
| GRA-TgAC9-F | GCAAGAAGCTTGATGGGATGGATATCATGGACGTCTCCGGTCGA | GRA-TgAC9-3×MYC |
| GRA-TgAC9-R | ATGAGTTTCTGCTCGATATCCATTCCCTGCGGATATTC |  |
| TUB-TgISP4-F | cccttttagatctaaaACTAGTATGGAAAGACGAAATCCA | TUB-TgISP4-3×MYC |
| TUB-TgISP4-R | GATGAGTTTCTGCTCGCTAGCTTCTCCGGTTGCTTTCTT |  |
| TgVPS13A-N928-1392-F | GTTGTCTTTGTCTCTTCCACGATGGAGTTCGTCGAGCAT | Pro-TgVPS13A-N928-1392-SmFP-MYC |
| TgVPS13A-N928-1392-R | TCCGATATCAGTTTTTGCTCGCTAGCCGTCGCGTGCAGAAGAGG |  |
| TgVPS13A-VAB-ATG2_C --F | ATGTTAGTGAACATTGAGGGCAGGAA | Pro-TgVPS13A-VAB-ATG2_C -SmFP-MYC |
| TgVPS13A-VAB-ATG2_C -R | CGATATCAGTTTTTGCTCGCTAGCATCTTCGTCTTGGTATTT |  |
| TgVPS13A-promoter-F | CCGCGGTGGCGGCCGCTCTAGAAGCAGGCAGTCTCTGTCT |  |
| TgVPS13A-promoter-R | TTCCTGCCCTCAATGTTCACTAACATCGTGGAAGAGACAAAGAC |  |
| TgSec13-C-gRNA-R | tatttctagctctaaaacCCGTTCTGCTCTTCCAGAGACaacttgacatccccattt | pSAG1-U6-TgSec13-C-Cas9 |
| TgSec13-C-3V5-F | GCGCCTCGGGCGCCGATGTATGCGCCTTACAAAGGAAACGGAAAACCTATACCGAAC | Fragment used for insertion of 3V5 tag into TgSec13 C terminal |
| TgSec13-C-3V5-R | CCTTCTCTCTTTTCTCACAGCCGTTCTGCTGTTCCAGAGATTAACCTTGGCCCGTGGA |  |
| TgSec13-C-Iden-F | CTGCGGCGAATTCCGTGACC | PCR identification of 3V5 insertion in TgSec13 C terminal |
| TgSec13-C-Iden-R | TTCTCCCCACAGTGCATGCG |  |
| TgDAT1-promoter-F | GCGGTGGCGGCCGCTCTAGAGCGGCCATGGCAAAGGTT | cwt:Pro-TgDAT1-SmFP-MYC |
| TgDAT1-Promoter-R | CACTTTCGTCGTAGTCctaGATATCTTCATTGGCAGAGCGAAA |  |
| TgDAT1-F | TTTTTCGCTCTGCCAATGAAACTAGTATGGAGACCTTCGCCCCC |  |
| TgDAT1-R | TCCGATATCAGTTTTTGCTCGAAAGAGAAGGTCGGAAG |  |
| TgDAT1-316D/N-326E/Q-F | CTTCTCATGTCGAGCTTGTGCAACTTCaACCACGGAGTGATTCCAGCCGTTCTGGGGcAACTTCAGGAACACTTCCCGCAGATGGCG | cmut:Pro-TgDAT1-mut-SmFP-MYC |
| TgDAT1-316D/N-326E/Q-R | CGCCATCTGCGGGAAGTGTTCCTGAAGTTgCCCCAGAACGGCTGGAATCACTCCGTGGTtGAAGTTGCACAAGCTCGACATGAGAAG |  |
| TgDAT1-397R/Q-F | GAGTCTCGCCGTCATGTACTTCACGCaaTTCTGCATTGGCCTTTGTCAGGCCT |  |
| TgDAT1-397R/Q-R | AGGCCTGACAAAGGCCAATGCAGAAttGCGTGAAGTACATGACGGCGAGACTC |  |
| TgDAT1-576K/C-579K/C-F | CACCGAGTATATGGTCGTGGTTCTCtgtTTCGGCtgtATCAACGTCGTCGTTCTCTCCACCC |  |
| TgDAT1-576K/C-579K/C-R | GGGTGGAGAGAACGACGACGTTGATacaGCCGAAacaGAGAACCACGACCATATACTCGGTG |  |
| TgDAT1-617R/Q-F | GCGGCTACCGAGGGGGGCAGCAAcaGATGGCGGTGCGGGTCGCAACGGT |  |
| TgDAT1-617R/Q-R | ACCGTTGCGACCCGCACCGCCATCtgTTGCTGCCCCCCTCGGTAGCCGC |  |
| TgDAT1-722E/Q-F | GCAGACCGCTTTCCCTGTCTCCAAGcAACTTCCTCTGGAAGTCGGCTTTTCGA |  |
| TgDAT1-722E/Q-R | TCGAAAAGCCGACTTCCAGAGGAAGTTgCTTGGAGACAGGGAAAGCGGTCTGC |  |
| PCAGGS-TgVPS13A-VAB-F | ggtaGCCACCATGGGTACCatgTTAGTGAACATTGAGGGC | PCAGGS-TgVPS13A_VAB-3×HA |
| PCAGGS-TgVPS13A-VAB-R | TCAGGAACATCGTATGGGTATGTGCCGGGCTTCGAGCG |  |
| PCAGGS-TgDAT1-F | tcgatgcataggtaGCCACCATGGAGACCTTCGCCCCC | PCAGGS-TgDAT1-3×FLAG |
| PCAGGS-TgDAT1-R | CATGGTCTTTGTAGTCGGTACCGAAAGAGAAGGTCGGAAG |  |
| PCAGGS-TgDAT1-△N-1-303-F | atgcataggtaGCCACCatgGTCTGCATGCTTCTCATGTC | PCAGGS-TgDAT1-△N-1-303-3×FLAG |
| PCAGGS-TgDAT1-△N-1-303-R | CATGGTCTTTGTAGTCGGTACCGAAAGAGAAGGTCGGAAG |  |
| PCAGGS-TgVAP-F | atcgatgcataggtaGCCACCATGGCGGGACCGTTGCTG | PCAGGS-TgVAP-3×FLAG |
| PCAGGS-TgVAP-R | ATGGTCTTTGTAGTCGGTACCCTTAGTGATTTCCGCCGA |  |
| PCAGGS-TgVPS13A-N929-1392aa-F | tcgatgcataggtaGCCACCATGTTCTCTCTCTCCAACTTT | PCAGGS-TgVPS13A-N928-1392aa-3×HA |
| PCAGGS-TgVPS13A-N929-1392aa-R | TCAGGAACATCGTATGGGTACGTCGCGTGCAGAAGAGG |  |
| PCAGGS-TgVPS13A-ATG2-C-F | atgcataggtaGCCACCATGCGCCAGGAACTCTGCTTT | PCAGGS-TgVPS13A-ATG2-C-3×HA |
| PCAGGS-TgVPS13A-ATG2-C-R | TCAGGAACATCGTATGGGTAATCTTCGTCTTGGTATTT |  |
| TgSEC61β-C-gRNA-R | tatttctagctctaaaacACTTCGAGCTCTGCTCCTCTaacttgacatccccattt | pSAG1-U6-TgSEC61β-C-Cas9 |
| TgSEC61β-C-3V5-F | CATCGTCGGCAAGGTTCATCAGACGTACGGCGGAGAGAACGGAAAACCTATACCGAAC | Fragment used for insertion of 3V5 tag into TgSEC61β C terminal |
| TgSEC61β-C-3V5-R | CAAAGATCTGAACGAGCTCgAACTgCGtGCTCTGCTCCTCTAACCTTGGCCCGTGGA |  |
| TgSEC61β-C-Iden-F | AGAGGACAACGCGTTCTCAC | PCR identification of 3V5 insertion in TgSEC61β C terminal |
| TgSEC61β-C-Iden-R | CCTGTTCATCGAGGTCGTCT |  |
| TgGAPM3-C-gRNA-R | ctatttctagctctaaaacGTGTCAACCTTGTCCCTTAGCaacttgacatccccattt | pSAG1-U6-TgGAPM3-C-Cas9 |
| TgGAPM3-C-EGFP-F | AGCACCCTTTCCGTTCGACTTTCCATGTTGCGTGCAGGCCgtgagcaagggcgaggag | Fragment used for insertion of EGFP tag into TgGAPM3 C terminal |
| TgGAPM3-C-EGFP--R | TTACGCTGACTGAAAAAACGAGTGTCGACCTTGTCCCTTAGATATCcttgtacagctc |  |
| TgGAPM3-C-Iden-F | GTCTCAGACTTCCATGCTGG | PCR identification of EGFP insertion in TgGAPM3 C terminal |
| TgGAPM3-C-Iden-R | AGCGCCATCGTGGCACTTCG |  |
| GRA-Lact-C2-GFP-F | AGCAAGAAGCTTGATGGGATGatggtgagcaagggcgag | GRA-Lact-C2-GFP |
| GRA-Lact-C2-GFP-R | CGTCGTACGGATACATCTAGCTAGCctaacagcccagcagctcc |  |
| TgGAPM3-C-Mcheery-F | AGCACCCTTTCCGTTCGACTTTCCATGTTGCGTGCAGGCCGTGAGCAAGGGCGAGGAG | Fragment used for insertion of Mcheery tag into TgGAPM3 C terminal |
| TgGAPM3-C-Mcheery-R | TTACGCTGACTGAAAAAACGAGTGTCGACCTTGTCCCTTACTTGTACAGCTCGTCCATG |  |

S2 Table. List of all primers used in this study. All primers were designed by Snapgene.
