# Supplementary material for: An ER-IMC bridge protein TgVPS13A and an IMC-resident scramblase TgDAT1 drive daughter budding in Toxoplasma gondii
Source: PLoS Pathog. 2026 Jun 18;22(6):e1013865. doi: 10.1371/journal.ppat.1013865 (PMC13298984; doi:10.1371/journal.ppat.1013865)
Supplement: S3 Table — The table presents the specific guide RNA sequences of TgVPS13A (TGGT1_291180), TgVAP (TGGT1_318160), TgDAT1 (TGGT1_258700), TgIMC29 (TGGT1_243200), TgGAPM3 (TGGT1_271970), TgSec61β (TGGT1_211040) and TgSec13 (TGGT1_201700). (DOCX) [file ppat.1013865.s011.docx]

**S3 Table**

| Gene ID | gRNA sequences |
| --- | --- |
| TGGT1_291180 | AATCGCCAGAGTCTCAGCGA |
|  | ATCACGCTCACGACCGCTGG |
|  | CCAACTGCCTAACTCGACCA |
| TGGT1_318160 | TGATTGAAACGCGAAAATGG |
| TGGT1_258700 | GGCGAAGGTCTCCATTTCAT |
| TGGT1_243200 | CCTTTAATTGAGGCCGTGTC |
| TGGT1_271970 | CTAAGGGACAAGGTTGACAC |
| TGGT1_211040 | AGAGGAGCAGAGCTCGAAGT |
| TGGT1_201700 | TCTCTGGAAGAGCAGAACGG |

S3 Table. The list of all guide RNA sequences in this study.

The table presents the specific guide RNA sequences of TgVPS13A (TGGT1_291180), TgVAP (TGGT1_318160), TgDAT1 (TGGT1_258700), TgIMC29 (TGGT1_243200), TgGAPM3 (TGGT1_271970), TgSec61β (TGGT1_211040) andTgSec13 (TGGT1_201700). Extract the 200bp sequences at the C or N terminus of these genes and input them into the Eukaryotic Pathogen CRISPR guide RNA/DNA Design Tool to select the optimal gRNA sequence for homologous recombination onto the pCD-Cas9 vector to construct the targeted Cas9 plasmid.
