# Supplementary material for: An ER-IMC bridge protein TgVPS13A and an IMC-resident scramblase TgDAT1 drive daughter budding in Toxoplasma gondii
Source: PLoS Pathog. 2026 Jun 18;22(6):e1013865. doi: 10.1371/journal.ppat.1013865 (PMC13298984; doi:10.1371/journal.ppat.1013865)
Supplement: S1 File — (DOC) [file ppat.1013865.s012.doc]

**Supporting methods**

**Co-immunoprecipitation**

For Co-immunoprecipitation, HEK293T cells at 70% confluency in 6cm dishes were transfected with plasmids, collected after 48 h, and lysed in RIPA buffer for 30 min on ice. The supernatant (100 μL) was used for input analysis. The remaining supernatant (300 μL) was incubated with Anti-FLAG M2 Magnetic Beads (Sigma-Aldrich, M8823) or anti-HA Affinity Gel (Sigma) for overnight at 4˚C with gentle rotation. The beads were washed five times with PBS and RIPA buffer, and bound proteins were eluted with SDS-PAGE sample buffer.

**Protein Structure Modelling and Molecular Docking**

TgVAP and TgDAT1 structures were modelled with Alphafold3 (1). TgVPS13 was modelled as three different parts (N terminus, Bridge, C terminus) and stitched together by Pymol (2). The stitched structure was manually curated to highlight the backbone of the protein with the lipid transfer bridge and the membrane binding domains on N and C terminus. Molecular docking of TgVPS13A with TgDAT1 and TgVAP was performed using HADDOCK (3, 4), and visualized by the Biovia Discovery Studio (5). The potential sites for salt bridge were predicted by the Chimera.

# References

1. Abramson J, Adler J, Dunger J, Evans R, Green T, Pritzel A, et al. Accurate structure prediction of biomolecular interactions with AlphaFold 3. Nature. 2024;630(8016):493-500.
2. Cai S, Wu Y, Guillén-Samander A, Hancock-Cerutti W, Liu J, De Camilli P. In situ architecture of the lipid transport protein VPS13C at ER-lysosome membrane contacts. Proc Natl Acad Sci U S A. 2022;119(29):e2203769119.

3. Honorato RV, Trellet ME, Jiménez-García B, Schaarschmidt JJ, Giulini M, Reys V, et al. The HADDOCK2.4 web server for integrative modeling of biomolecular complexes. Nat Protoc. 2024;19(11):3219-41.

4. Honorato RV, Koukos PI, Jiménez-García B, Tsaregorodtsev A, Verlato M, Giachetti A, et al. Structural Biology in the Clouds: The WeNMR-EOSC Ecosystem. Front Mol Biosci. 2021;8:729513.

5. BIOVIA, Dassault Systèmes, Discovery Studio visualizer v25.1.0.24284, Release 2025, San Diego: Dassault Systèmes, 2025.
